# The human CD47 checkpoint is targeted by an immunosuppressive *Aedes aegypti* salivary factor to enhance arboviral skin infectivity

**DOI:** 10.1126/sciimmunol.adk9872

**Published:** 2024-08-09

**Authors:** Alejandro Marin-Lopez, John D. Huck, Allen T. Esterly, Veronica Azcutia, Connor Rosen, Rolando Garcia-Milian, Esen Sefik, Gemma Vidal-Pedrola, Hamidah Raduwan, Tse-Yu Chen, Gunjan Arora, Stephanie Halene, Albert C. Shaw, Noah W. Palm, Richard A. Flavell, Charles A. Parkos, Saravanan Thangamani, Aaron M. Ring, Erol Fikrig

**Affiliations:** 1 Section of Infectious Diseases, Department of Internal Medicine, Yale University School of Medicine, New Haven, CT, USA.; 2 Department of Immunobiology, Yale University School of Medicine, New Haven, CT, USA.; 3 Department of Microbiology and Immunology, State University of New York, Upstate Medical University, Syracuse, NY, USA.; 4 Department of Pathology, University of Michigan School of Medicine, Ann Arbor, MI, USA.; 5 Bioinformatics Support Program, Cushing/Whitney Medical Library, Yale School of Medicine, New Haven, CT, USA.; 6 Section of Hematology, Department of Internal Medicine, Yale University School of Medicine, New Haven, CT, USA.

## Abstract

The *Aedes aegypti* mosquito is a vector of many infectious agents, including flaviviruses such as Zika virus. Components of mosquito saliva have pleomorphic effects on the vertebrate host to enhance blood feeding, and these changes also create a favorable niche for pathogen replication and dissemination. Here, we demonstrate that human CD47, which is known to be involved in various immune processes, interacts with a 34-kilodalton mosquito salivary protein named Nest1. Nest1 is up-regulated in blood-fed female *A. aegypti* and facilitates Zika virus dissemination in human skin explants. Nest1 has a stronger affinity for CD47 than its natural ligand, signal regulatory protein α, competing for binding at the same interface. The interaction between Nest1 with CD47 suppresses phagocytosis by human macrophages and inhibits proinflammatory responses by white blood cells, thereby suppressing antiviral responses in the skin. This interaction elucidates how an arthropod protein alters the human response to promote arbovirus infectivity.

## INTRODUCTION

Blood-feeding arthropods serve as vectors for some of the most devastating global infectious diseases. Each year, mosquito-borne diseases afflict more than 700 million people worldwide, leading to more than 1 million deaths (1, 2). Mosquito-borne viruses are maintained in both an arthropod vector and a vertebrate host, and transmission from an infected mosquito to a susceptible host is a critical point in the viral life cycle (3). Mosquitoes maintain viruses in various tissues, including their salivary glands. When they bite, saliva is secreted into the vertebrate host to facilitate blood feeding and digestion, and it contains proteins with angiogenic, anti-inflammatory, and other immunomodulatory properties that counteract host hemostasis (4). For example, a mosquito apyrase (5) alters platelet aggregation; aegyptin inhibits collagen-induced clot formation (6, 7); D7 proteins scavenge biogenic amines, affecting physiological effectors such as norepinephrine, serotonin, histamine, and eicosanoids (8, 9); and sialokinin disrupts endothelial integrity (10).

During a bite, viruses are also introduced with saliva into the host skin. The mosquito bite itself and the saliva inoculated during feeding can both modulate the local skin immune response, thereby influencing viral infectivity (11–15). D7 protein inhibits dengue virus infection (16), whereas a factor Xa–directed anticlotting serpin-like protein and an adenosine deaminase increase the dengue viral burden in human epidermal keratinocytes in vitro (17). In addition, the salivary protein LTRIN (lymphotoxin β receptor inhibitor) enhances Zika virus (ZIKV) replication by interacting with LTβR (lymphotoxin β receptor), thereby inhibiting NF-κB (nuclear factor κB) pathways (18). We have recently shown that AgBR1 (*Aedes aegypti* bacteria-responsive protein 1) and Nest1 (neutrophil-stimulating factor 1) are two salivary antigens that enhance ZIKV dissemination in the AG129 mouse model (19, 20). However, the specific pathways by which these salivary proteins mediate the infectivity of arboviruses remain largely unknown. Thus, elucidating the mechanisms by which vector factors interact with human ligands may lead to new approaches to control or prevent deadly arthropod-borne diseases.

Here, we demonstrate that the expression of the *A. aegypti* salivary protein AAEL003601, also named Nest1, is up-regulated in blood-fed female mosquitoes and facilitates virus dissemination in human skin explants. Furthermore, Nest1 interacts with human CD47, which is an immune checkpoint molecule with pleiotropic activity (21) and is overexpressed in multiple solid and hematological cancers (22–24). CD47 is known to be expressed in multiple cells types, such as hematopoietic cells, including thymocytes, T and B cells, monocytes, platelets, and erythrocytes, as well as on epithelial, endothelial cells, among others (25). CD47 regulates phagocytosis in macrophages through its interaction with signal regulatory protein α (SIRPA) and integrins (26, 27) and has additional roles in erythrocyte homeostasis and clearance of infected cells (28–31). CD47 and SIRPA also modulate neutrophil migration across endothelial and epithelial monolayers (32, 33). Moreover, CD47 interacts in cis with CD11b/CD18 to regulate polymorphonuclear cell transepithelial migration in vivo (34). Under some conditions, CD47 ligation induces T cell proliferation (35), but CD47 can also suppress T cell activation via thrombospondin 1 (36). In this work, we show that Nest1 alters CD47-mediated phagocytosis, inhibits proinflammatory responses in white blood cells, and triggers virus dissemination in the human skin, thus down-regulating immune pathways and antiviral responses and triggering the expression of viral replication and infection-related genes. Understanding the role of human cutaneous responses against *A. aegypti* antigens represents a critical area of study because of the consequences of these interactions in the spread of mosquito-borne diseases.

## RESULTS

### *A. aegypti Nest1* is expressed in salivary glands and induced after blood feeding

**Fig. 1.**
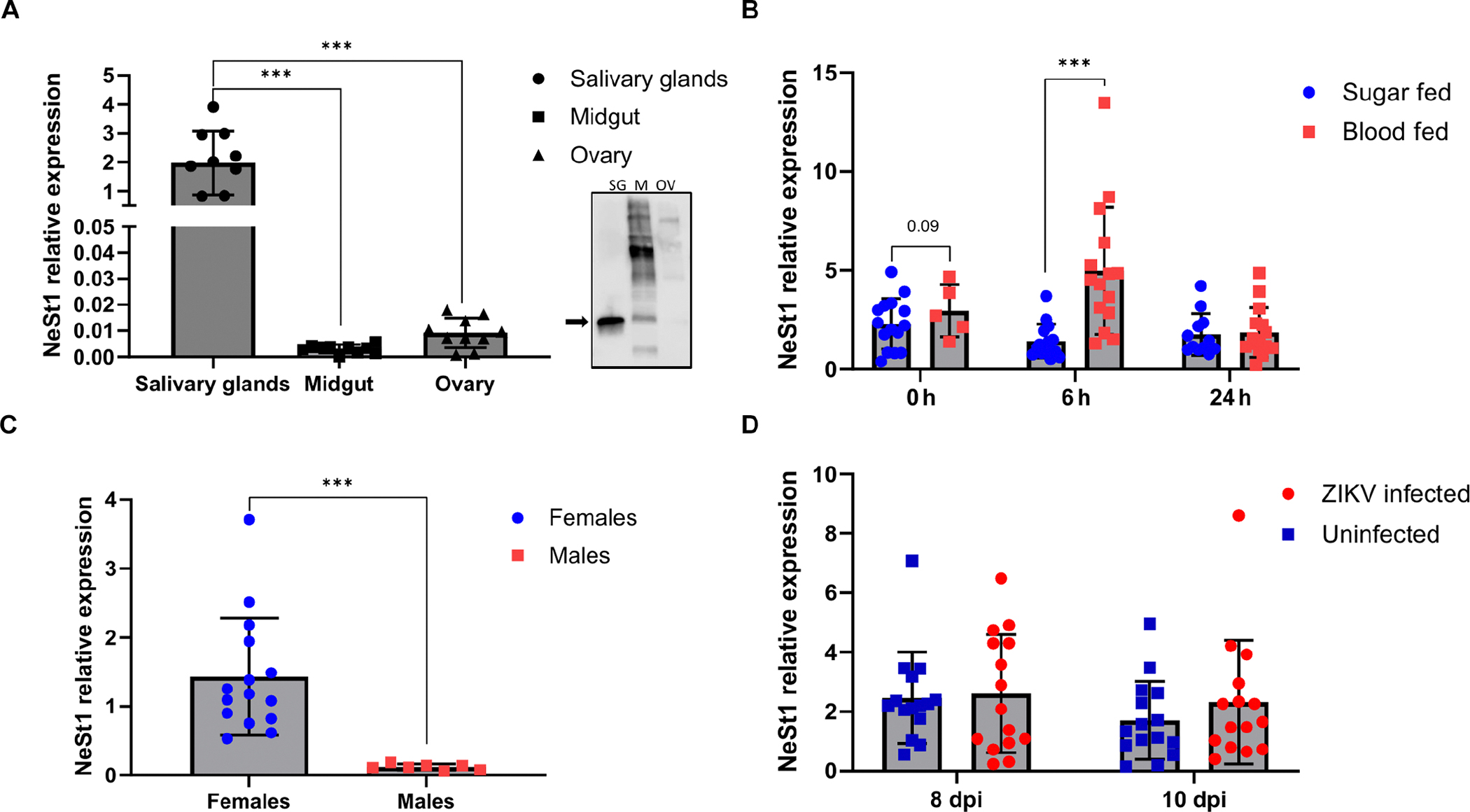
Characterization of *Nest1* expression in the mosquito. (**A**) *Nest1* relative expression in salivary glands (SG), midgut (M), and ovaries (OV) of female *Aedes aegypti* by qRT-PCR and protein immunoblot. (**B**) *Nest1* relative expression in salivary glands after sugar or blood meal at 0 (immediately after), 6, and 24 hours after feeding by qRT-PCR. (**C**) *Nest1* relative expression in salivary glands in male and female mosquitoes after a sugar meal. (**D**) *Nest1* relative expression after ZIKV intrathoracic infection. Relative expression of *Nest1* 8 and 10 dots per inch (dpi) in ZIKV-infected and noninfected mosquitoes. Ten to 15 mosquitoes per group were used in every experiment. qRT-PCR relative expression was analyzed using *Rp49* as a housekeeping gene. These values represent the means ± SEM from a single experiment and are representative of two independent experiments. The *P* value is displayed in the graph and was determined using Student’s *t* test. Asterisks represent significant difference between samples (***P < 0.001).

We characterized the expression of the gene encoding *Nest1* (AAEL00 3601) and Nest1 protein in different mosquito tissues by quantitative reverse transcription polymerase chain reaction (qRT-PCR) or protein immunoblot. *Nest1* expression was most prominent in the salivary glands and evident in the midgut and ovaries (Fig. 1A). Then, we analyzed *Nest1* expression in natural blood-fed and sugar-fed female mosquitoes. *Nest1* expression was significantly induced in the salivary glands of fully engorged blood-fed mosquitoes 6 hours after feeding (*P* < 0.0001), relative to its expression in sugar-fed mosquitoes, returning to similar values 24 hours after feeding (Fig. 1B). Differences in *Nest1* expression were also examined in male and female mosquito salivary glands, with a significantly higher relative expression in female salivary glands (*P* < 0.0001) (Fig. 1C).

To test whether *Nest1* expression was controlled by neuroolfactory regulation, mesh-vented cups containing female mosquitoes were caged in the presence of mice for 30 min. No differences were observed in the *Nest1* transcripts between stimulated and unstimulated (not caged in the presence of mice) mosquitoes under these conditions (fig. S1A). We also determined the amount of Nest1 protein present in the salivary glands and its concentration in the saliva. By protein immunoblot, we quantified the intensity of the Nest1 band in several salivary gland pairs, using a standard of known concentrations of recombinant Nest1 (rNest1) and rabbit anti-Nest1 antibodies to specifically detect Nest1. We estimated an average amount of 47 ng per pair of glands (fig. S1B). Previous works demonstrated that approximately half of the protein content in the salivary glands is discharged during a blood meal, and between 2 and 1000 nl of saliva can be secreted (37–41). On the basis of these quantifications, we estimated a concentration of Nest1 in mosquito saliva ranging between 1 and 350 μM (0.71 to 349 μM).

We also tested whether virus infection altered the expression of *Nest1* in the salivary glands. The presence of ZIKV in mosquitoes did not influence *Nest1* expression (Fig. 1D and fig. S1C). In addition, amino acid sequence alignment analysis revealed Nest1 homologs in other hematophagous mosquito species, including *Aedes albopictus* (62.31%), *Armigeres subalbatus* (64.95%), *Culex pipiens pallens* (22.84%), *Culex quinquefasciatus* (25.66%), and *Uranotaenia lowii* (25.37%) (fig. S2, A and B). As an example, *A. aegypti* Nest1 antibodies bound a recombinant version of *C. quinquefasciatus* 34k2 protein (EDS43044.1; lane 1) and to a 34-kDa protein in *C. quinquefasciatus* salivary gland extracts (lanes 2 to 5) (fig. S2C). Together, these findings suggest an important role for Nest1 after mosquito blood feeding, potentially in multiple hematophagous mosquito vector species.

### Nest1 enhances viral replication in human skin explants

**Fig. 2.**
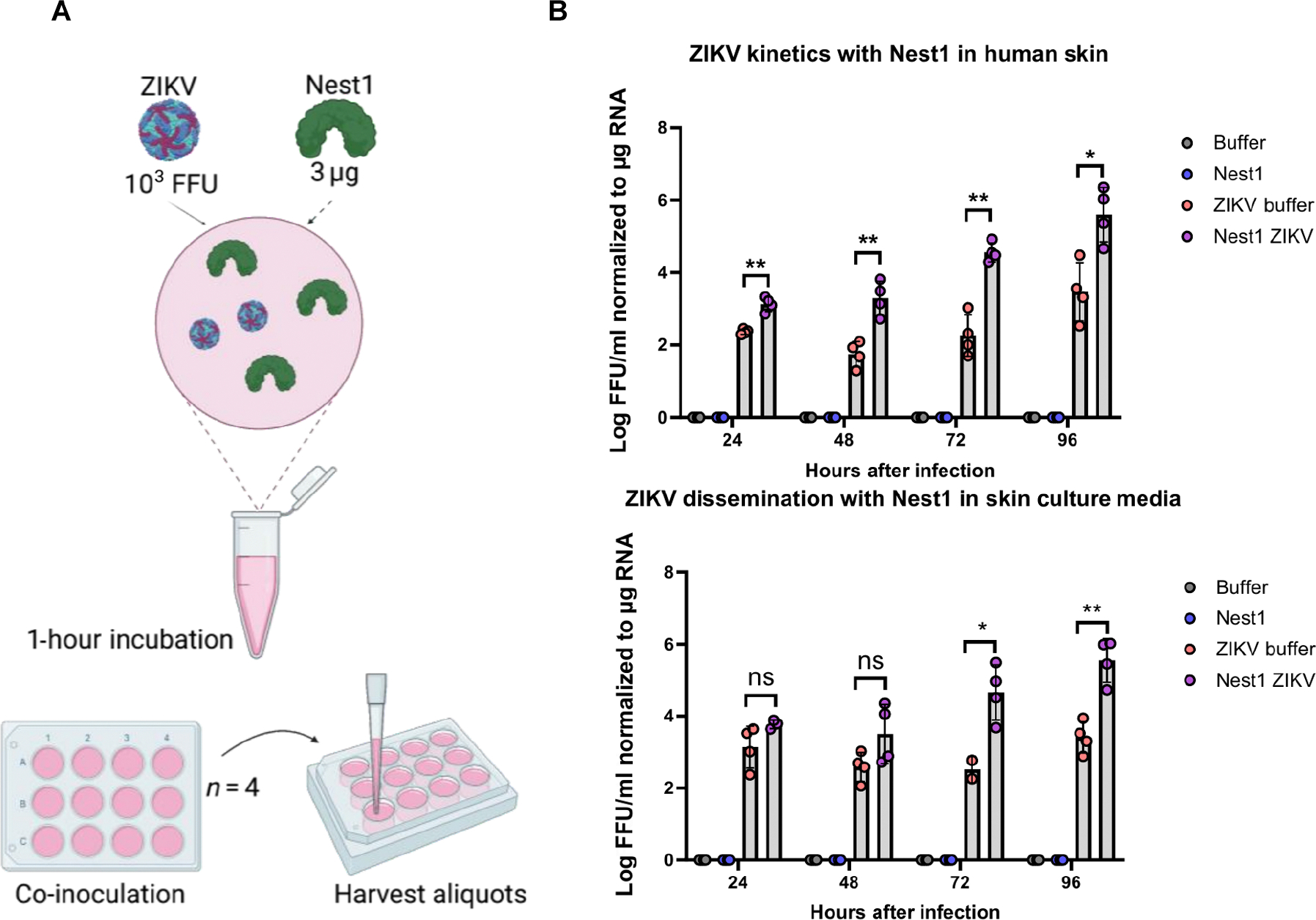
Nest1-ZIKV replication kinetics in the human skin. (**A**) One thousand FFUs of ZIKV Mex I44 and 3 μg of Nest1 protein were combined, incubated for 1 hour, and intradermally inoculated into the human skin (*n* = 4) for each group. Groups included (i) buffer, (ii) Nest1, (iii) ZIKV + buffer, and (iv) ZIKV + Nest1. Buffer (150 mM NaCl and 10 mM Hepes) in which Nest1 protein was collected was used as a negative control. (**B** and **C**) Graphs represent ZIKV FFU in skin samples (B) and culture media samples (C) in 24-hour intervals until 4 days after infection. Quantification of viral loads was calculated by real-time qRT-PCR using a standard curve in which FFUs were normalized to μg RNA by isolating RNA from a known high-titer ZIKV stock. The standard curve was generated by plotting log_10_ FFUs (from the known titer virus) as a function of Ct value obtained. Sample Ct values were applied to the standard curve to calculate the log_10_ FFUs, which were normalized to μg of RNA to account for variations in total RNA mass from different skin samples. These values represent the means ± SEM from a single experiment. The *P* value is displayed in the graph and was determined using Student’s *t* test. Asterisks represent significant difference between samples (*P < 0.05 and **P < 0.01). ns, not significant.

The initial interface between arboviruses and mosquito salivary proteins with the human host occurs in the skin. Characterizing the triangular human cell–mosquito salivary antigen–pathogen interactions in the context of complex tissue cytoarchitecture is critical for deciphering the mechanisms of many immune and pathogenic processes (42). Although sometimes useful, in vitro cell assays do not provide an adequate environment to study intricate interactions among viral, mosquito, and host factors, and murine models often do not recapitulate human disease. The role of Nest1 in human infection remains unknown. To test the effect of Nest1 on arboviral infection, we used an ex vivo experimental model of infection with human skin explants (Fig. 2A). Skin explants were intradermally inoculated with Nest1 or the buffer used to elute Nest1 as a control (buffer) in the absence (internal negative control for infection) or presence of ZIKV. In the presence of Nest1 protein, we observed an increase in ZIKV titers over time, from 24 to 96 hours after infection in the skin tissue and from 72 to 96 hours after infection in the supernatants collected from the explants (Fig. 2, B and C). This enhanced effect of a specific mosquito salivary protein on viral replication using human skin explants indicates that Nest1 may facilitate ZIKV infectivity at the inoculation site in humans.

### *A. aegypti* Nest1 interacts with human CD47

**Fig. 3.**
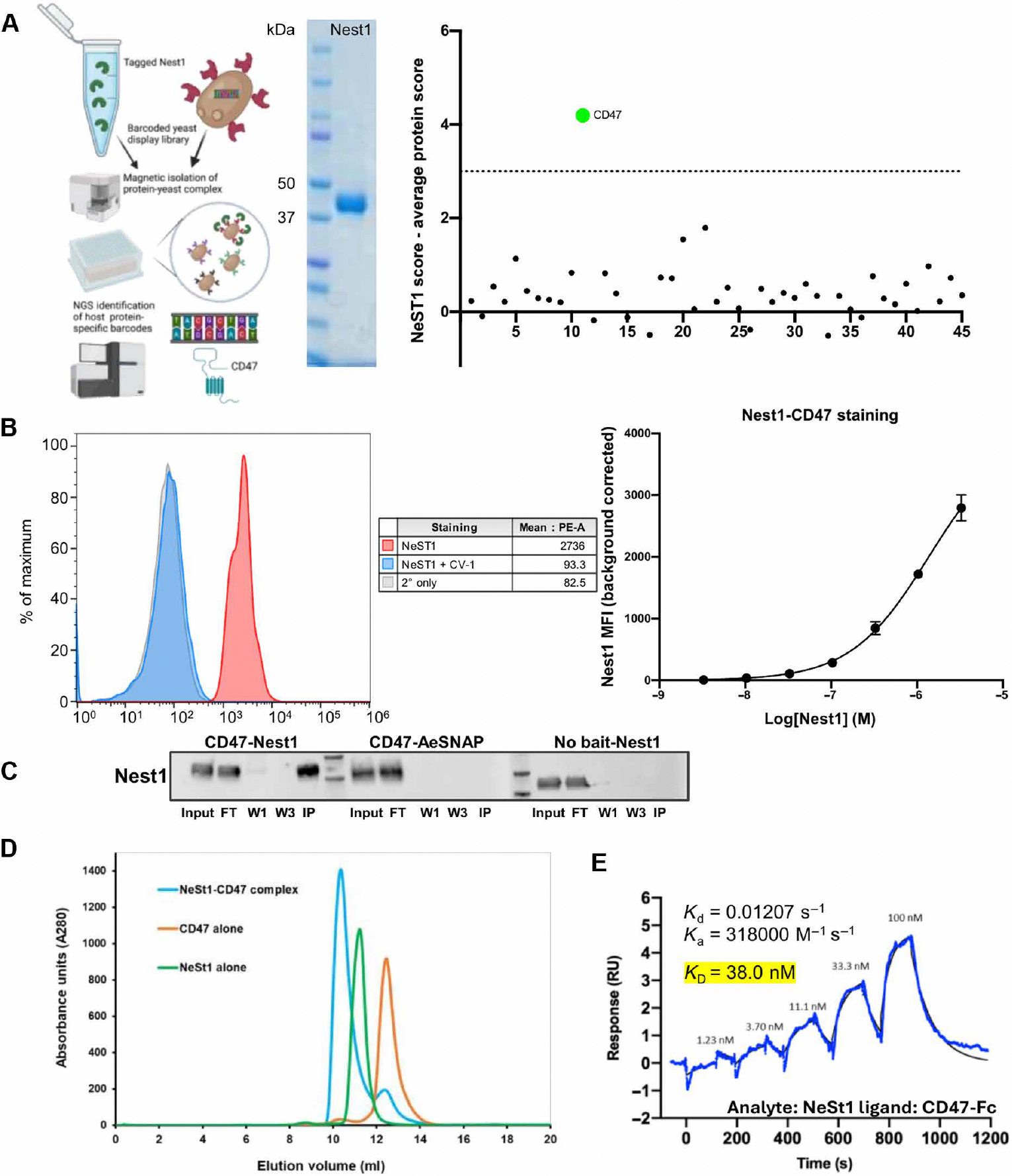
Characterization of the Nest1-CD47 interaction. (**A**) REAP diagram for the identification of protein-protein interactions. Schematic of the yeast-display screen. A library of yeast cells, each displaying a single human protein encoded by a uniquely barcoded plasmid, was pooled and mixed with surface biotinylated Nest1 expressed in Expi293F cells. The REAP library was scaled to a 96-well magnetic separation format for this screen. Magnetic separation using streptavidin microbeads followed by NGS was used to identify yeast displaying proteins that bound to the Nest1 protein. Plasmid DNA was isolated and sequenced to identify the proteins. The Nest1 REAP screening identified human CD47 as a potential binding candidate. The score for each gene is defined as the overall enrichment for that gene (relative to the unselected library) multiplied by the percentage of barcodes associated with the gene that was enriched (defined as logFC > 0). (**B**) Mean fluorescence intensity (MFI) for the CD47-Nest1 interaction (red peak) and inhibition of Nest1-CD47 binding by the SIRPA variant CV-1 (blue peak) (left) and MFI values under increasing concentrations of rNest1 protein (right). (**C**) Representative immunoblot showing coimmunoprecipitation assay of Fc-tagged human CD47 with Nest1. Nest1 presence was analyzed in input, flow-through (FT), first wash (W1), last wash (W3), and immune-precipitated protein (IP) using rabbit anti-Nest1 serum and anti-rabbit HRP antibody. AeSNAP mosquito protein as prey was used as a negative control as well as the absence of CD47 bait incubated with Nest1. (**D**) SEC analysis for the Nest1-CD47 interaction. The Nest1-CD47 complex (blue peak) was formed by incubating 1.2× molar excess of CD47 with Nest1 and incubated at RT for 45 min before injecting onto an ENrich SEC 70 column (Bio-Rad). Equivalent molar amounts of CD47 and Nest1 were injected separately to compare with the elution volume of the complex. (**E**) SPR analysis of the Nest1-CD47 interaction. Single-cycle kinetics and affinity measurements were performed on a BIAcore T200 instrument using a Biotin CAPture kit (Cytiva). RU, response units.

To explore the potential role of Nest1 in the interplay between the human host and the vector, we next used an ultrahigh-throughput interaction screening platform termed REAP (rapid extracellular antigen profiling) to identify a potential human receptor for Nest1 (43). Nest1 was biotinylated and panned against a genetically barcoded library of more than 2600 human extracellular and secreted proteins individually displayed on the surface of yeast. Yeast clones that bound to Nest1 protein were isolated by magnetic separation using streptavidin microbeads, and their specific barcode sequences were identified by next-generation sequencing (NGS) (Fig. 3A). Nest1 was biotinylated and screened for binding partners. As shown in Fig. 3A, only one clone expressing CD47, a ubiquitously expressed integrin-associated glycoprotein, bound to Nest1 with a score that far exceeded a stringent significance threshold. The interaction between Nest1 and yeast-displayed CD47 was then reproduced by flow cytometry (Nest1 peak in red; control peak in gray) in a dose-specific manner (Fig. 3B).

A series of orthogonal binding studies was performed to confirm the direct interaction between Nest1 and human CD47. First, protein complex immunoprecipitation demonstrated that CD47 could interact with Nest1 (Fig. 3C). Nest1 was detected by Nest1-specific antibodies in the elution of the CD47-Nest1 coimmunoprecipitation, in contrast with the negative controls used in this study (AesNAP mosquito protein or the absence of Nest1 Bait protein). The interaction was then analyzed using size exclusion chromatography (SEC), in which CD47 alone, Nest1 alone, or Nest1 incubated with a molar excess of CD47 was injected onto a gel filtration column and their elution volumes were measured. As shown in Fig. 3D, a higher–molecular weight species corresponding to the Nest1-CD47 complex was observed compared with the individual injections of Nest1 and CD47. Last, an affinity measurement for this interaction was performed using surface plasmon resonance (SPR). Nest1 bound strongly to CD47 with an equilibrium dissociation constant (*K*_d_) of ~38.0 nM (Fig. 3E). This affinity is more than 26 times higher than that for the SIRPα allele 2 (the most predominant SIRPA variant in human), a major human protein that interacts with CD47 (*K*_d_ ~ 1 μM) (44–46).

**Fig. 4.**
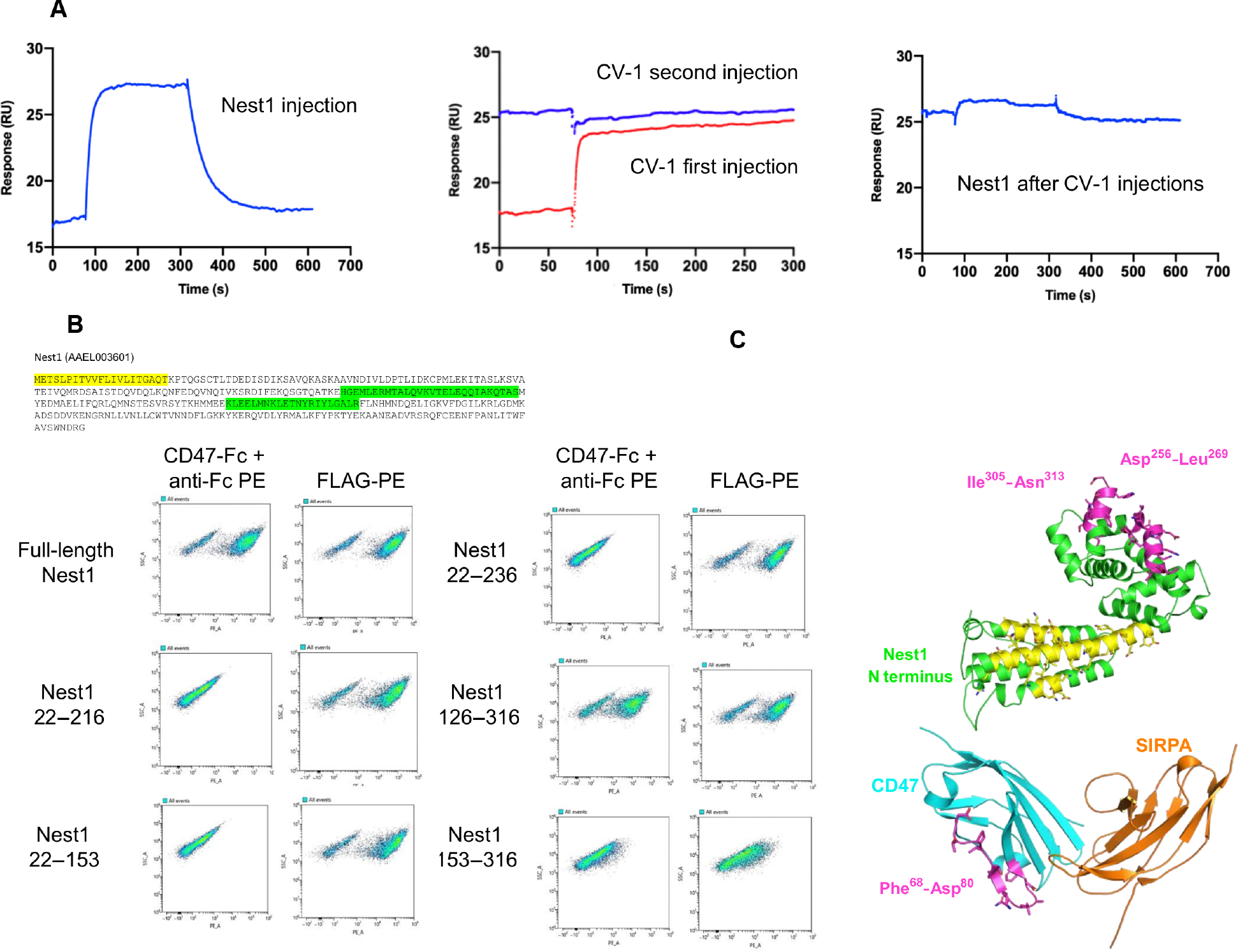
Biophysical characterization of the Nest1-CD47 interaction. (**A**) The Nest1-CD47 interaction is abrogated in the presence of CV-1 by SPR. Nest1 (100 nM) was flowed over immobilized CD47-Fc (biotinylated) on a Biotin CAPture chip and allowed to dissociate completely (left). Two sequential injections of 50 nM CV-1 were then used (red trace followed by blue trace) to saturate CD47 (middle) without any measurable dissociation, followed by a 10 nM injection of Nest1 (right). (**B**) Amino acid sequence of Nest1 protein, secretion signal peptide (yellow), and predicted coiled-coil domains (green) are shown (Eukaryotic Linear Motif resource) (top). CD47-Fc binding to yeast expressing different Nest1 constructs was measured by flow cytometry (bottom). The C-terminal region (amino acids 126 to 316) of Nest1 protein is responsible for the Nest1-CD47 interaction. FLAG-PE measures the display of Nest1 on yeast. (**C**) Structural modeling illustrating interaction epitopes identified for Nest1 and CD47 by HDX-MS. Epitope segments with lower and higher hydrogen-deuterium exchange are highlighted in pink and yellow, respectively, relative to uncomplexed Nest1 and CD47. Images were generated using PyMOL. Nest1 was modeled using the crystal structure of LIPS-2 [Protein Data Bank (PDB) 7TDR]. The CD47:SIRPA coordinates are from PDB 2JJS.

To determine whether SIRPA and Nest1 compete for the same binding surface on CD47, we performed competition assays using an engineered SIRPA variant, CV-1, which binds to CD47 with an affinity of 11.1 pM (47). Using flow cytometry and SPR, we found that the interaction between CD47 and Nest1 is completely blocked by CV-1 (Figs. 3B and 4A). By flow cytometry, CV-1 totally abrogated Nest1 binding to CD47-expressing yeast. By SPR, we tested Nest1-CD47 transient binding (Fig. 4A, left). After addition of CV-1, CD47 was saturated (Fig. 4A, middle) and blocked Nest1-CD47 binding (Fig. 4A, right), indicating that Nest1 and SIRPA share at least a partially overlapping epitope on CD47. To further characterize the Nest1-CD47 interaction interface, we generated truncation constructs of Nest1 on the basis of secondary structure analysis and displayed on the surface of the yeast. Among the constructs generated, only Nest1_126–316_ retained the same binding capacity as full-length Nest1, indicating that the N-terminal region of the Nest1 protein (amino acids 1 to 126) is dispensable for Nest1-CD47 binding (Fig. 4B). Therefore, the C terminus (amino acids 126 to 316) of Nest1, in which two coiled-coil domains are predicted (amino acid sequences 124 to 154 and 184 to 205, the Eukaryotic Linear Motif resource), is both necessary and sufficient for the interaction. Hydrogen-deuterium exchange mass spectrometry (HDX-MS) confirmed these results, indicating that amino acids 256 to 269 and 305 to 313 have a lower rate of exchange with deuterium when complexed with CD47, indicating that they become buried upon complex formation (Fig. 4C and fig. S3). The N terminus of Nest1, on the other hand, has multiple regions with increased HDX (Fig. 4C, yellow), which might indicate that Nest1 undergoes a conformational change upon CD47 binding to expose that region to a solvent. In line with our blocking studies using CV-1, amino acids 68 to 80 of CD47, which are involved in the SIRPA interaction, become buried upon Nest1 binding. Together, these results show that *A. aegypti* Nest1 forms a high-affinity complex with CD47 and can compete with SIRPA, suggesting that Nest1 might modulate biological functions governed by CD47.

### Nest1 inhibits phagocytosis and proinflammatory responses

Macrophages play an important role during the initial stages of arboviral infection in the skin, mediating phagocytosis and destruction of microorganisms and stimulating the action of other immune cells. CD47 has a critical role in macrophage-mediated phagocytosis by its interaction with SIRPA or CD11b/CD18, located on the macrophage surface (28, 48, 49). We therefore examined the role of Nest1 in phagocytosis.

**Fig. 5.**
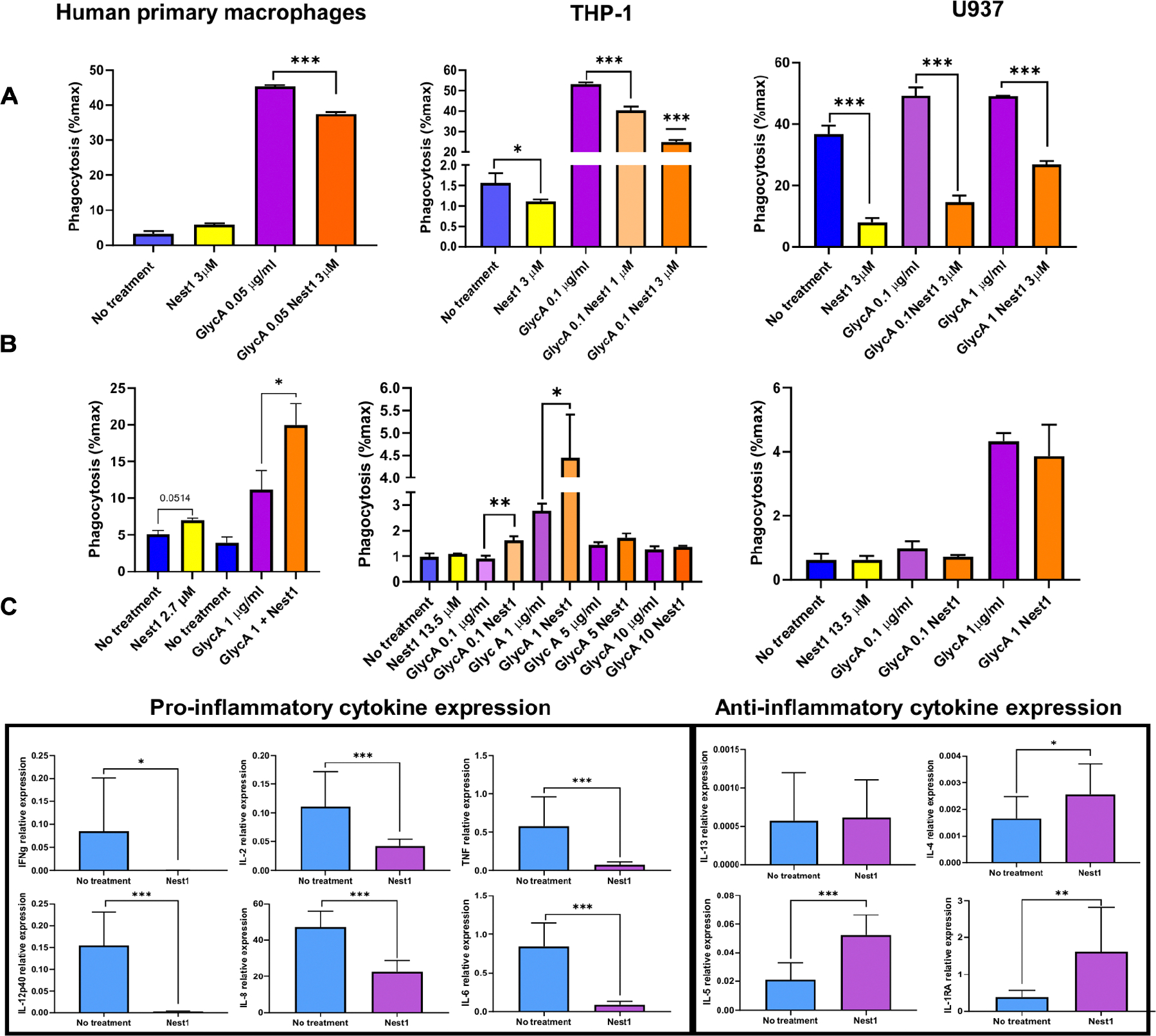
Phagocytosis assay of RBCs by different human immune cells and relative expression of proinflammatory cytokines in white blood cells. (**A** and **B**) Blood-derived human primary macrophages and macrophage-like THP-1 and U937 human cell lines were used in these experiments. Phagocytosis activity was measured 4 hours after stimulation (A). Phagocytosis assay with Nest1 and GlycA-preincubated RBCs (B). Blue bars, no treatment; yellow bars, Nest1 treatment; purple bars, GlycA); orange bars, Nest1+GlycA. These values represent the means ± SEM of three replicates from a single experiment. The *P* value is displayed in the graph and was determined using Student’s *t* test. Experiments with THP-1 and human primary macrophages are representative of two or three independent experiments. (**C**) Relative expression of proinflammatory cytokines (IFN-γ, IL-2, TNF, IL-12p40, IL-8, and IL-6) and anti-inflammatory cytokines (IL-13, IL-4, IL-5, and IL-1RA) from human white blood cells from four different donors. Expression was measured by qRT-PCR in nontreated and Nest1-treated human white blood cells 24 hours after stimulation. Cells were stimulated in triplicate, and the measurements of four different donors were pooled. Human GAPDH was used as a housekeeping gene. These values represent the means ± SEM of three replicates from a single experiment. Asterisks represent significant difference between samples, calculated by Student’s *t* test (*P < 0.05, **P < 0.01, and ***P < 0.001).

We first examined CD47 expression on the cell surface (fig. S4) by confocal microscopy imaging, observing high CD47 expression mainly in U937 and primary macrophages but also in THP-1 cells. To test whether Nest1 modifies macrophage phagocytosis, we performed in vitro assays with the promonocytic THP-1 and U937 cell lines, as well as blood-derived human primary macrophages, using human carboxyfluorescein succinimidyl ester (CFSE)–labeled red blood cells (RBCs) as target cells. On the basis of the Nest1 quantifications described above (24 ng of Nest1 secreted in approximately 250 nl of saliva; concentration of 2.84 μM), we performed our initial phagocytosis experiments using 3 μM Nest1 protein. Unexpectedly, the presence of Nest1 reduced the phagocytic activity of both cell lines and the primary cells (Fig. 5A). Basal phagocytic activity was reduced in both THP-1 and U937 cells after Nest1 treatment [yellow bars compared with control bars (blue)]. Opsonin-enhanced phagocytosis [glycophorin A (GlycA)] was also decreased in the presence of Nest1 (purple bars compared with orange bars). A possible explanation for this finding is that Nest1 can disrupt the cis CD47 interactions with other integrins such as CD18/CD11b on the surface of the macrophages, which have been shown to enhance phagocytosis (26). To test whether this inhibitory effect was due to the Nest1-CD47 macrophage interaction, we performed an additional phagocytosis assay in which we preincubated the target RBCs for 1 hour in the presence of Nest1 and washed them before adding the phagocytic cells. With this approach, we aimed to prevent direct interaction between Nest1 and the CD47 molecules expressed on the surface of the macrophages, only allowing triggering of Nest1 interactions with CD47 expressed on RBCs. Under these conditions, we observed a reversion of phagocytosis inhibition in primary human macrophages, THP-1 and U937 cells (Fig. 5B). Together, these results suggest that Nest1 can inhibit phagocytosis activities through the CD47 receptor on the surface of phagocytic cells. Additional data also showed that some polymorphonuclear neutrophil (PMN) immune functions related to CD47, including chemotaxis and CD11b surface activation, were inhibited in the presence of Nest1 (fig. S5, A to C).

During infection, immune cells produce proinflammatory molecules, such as cytokines and chemokines, to help the host to clear pathogens. Mosquito saliva can be detected in the skin even at 18 hours after bite (50). Therefore, we also examined the effect of Nest1 on the production of proinflammatory and anti-inflammatory mediators by white blood cells at an early stage. The expression of transcripts encoding proinflammatory molecules, including interferon-γ (IFN-γ), interleukin-2 (IL-2), tumor necrosis factor (TNF), and IL-12p40, were inhibited in cells treated with Nest1 after 24 hours (Fig. 5C). Neutrophil chemoattractants, including IL-8 and mediators of the acute phase response such as IL-6, were also down-regulated. In contrast, Nest1 did not influence or up-regulate the expression of anti-inflammatory mediators such as IL-13 or IL-4, IL-5, and IL1-RA, respectively (Fig. 5C). The cytokine IL-10 was found to be down-regulated in the presence of Nest1, whereas the monocyte chemoattractant MCP-1 (also called CCL-2) was up-regulated (fig. S5D), supporting our previous data analyzing murine neutrophils (20). Together, these data show that Nest1 inhibits proinflammatory responses and induces anti-inflammatory responses in white blood cells.

### Integrated analysis of Nest1 cutaneous transcriptional signatures shows immune and antiviral gene suppression (RNA sequencing)

**Fig. 6.**
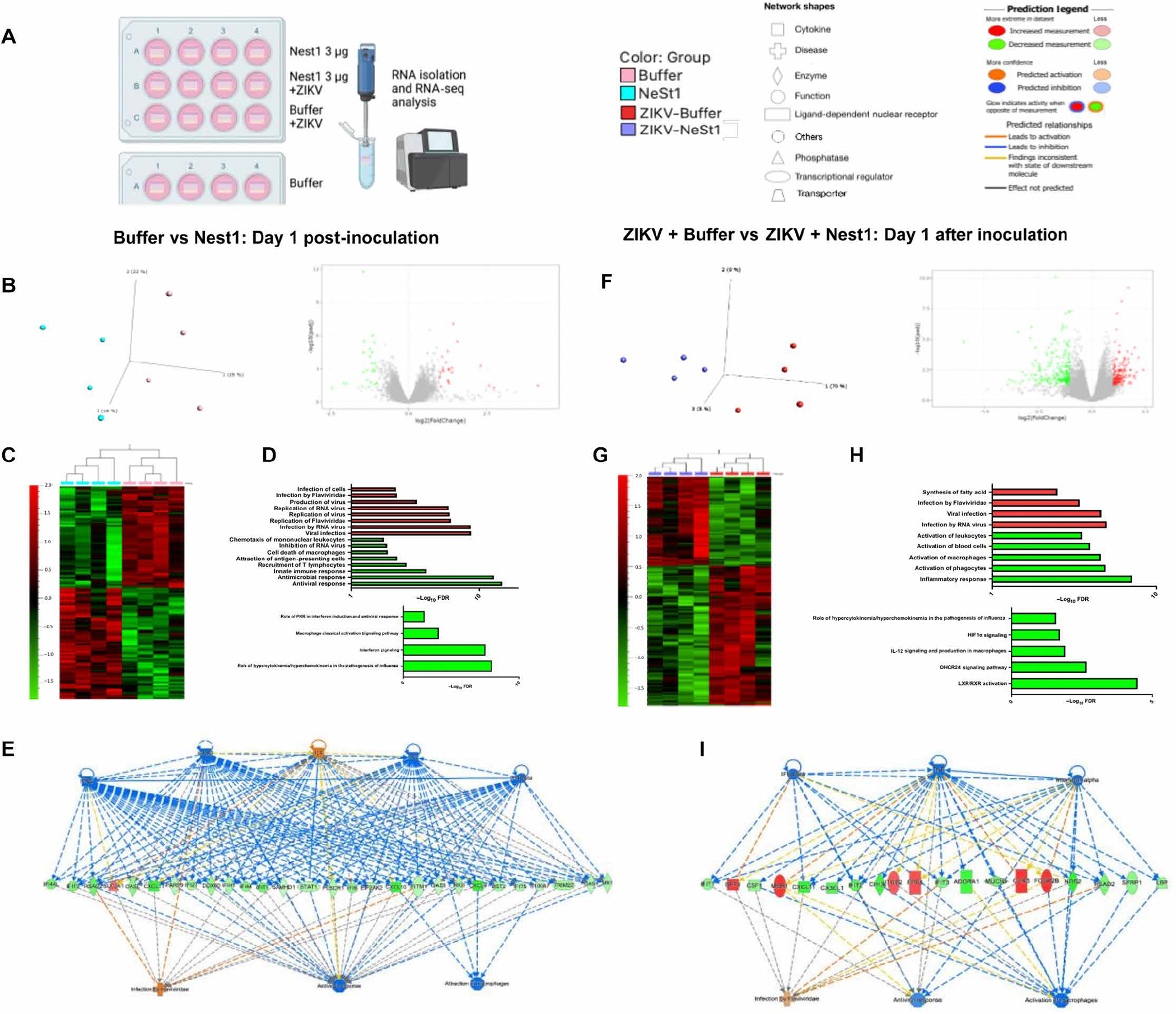
Transcriptomic analysis in human skin samples treated with buffer, Nest1, ZIKV (ZIKV buffer), and Nest1+ZIKV (ZIKV Nest1). (**A**, **B**, **F**, and **G**) Principal components assays and volcano plots for the DEGs after Nest1 and ZIKV+Nest1 treatments compared with control buffer and ZIKV buffer, respectively. The gene names can be found in data file S1. PCA reveals the similarity between samples based on the distance matrix. Samples were projected to a 3D plane spanned by their first three principal components. The percentage of the total variance per direction is shown in the label. The global transcriptional change across the groups compared was visualized by volcano plots. Each data point in the scatter plot represents a gene. The log_2_ FC of each gene is represented on the *x* axis versus the −log_10_ of its adjusted *P* value on the *y* axis. Genes with an adjusted *P* value less than 0.05 and a log_2_ FC greater than 1 are indicated by red dots. These represent up-regulated genes. Genes with an adjusted *P* value of less than 0.05 and a log_2_ FC less than 1 are indicated by green dots (down-regulated genes). RNA-seq, RNA sequencing. (**C** and **H**) A biclustering heatmap was used to visualize the expression profile of the top 30 DEGs sorted by their adjusted *P* value by plotting their log_2_ transformed expression values in samples. (**D** and **I**) Significant DEGs were clustered by their gene ontology (functions, top; pathways, bottom). Red bars represent up-regulated functions. Green bars represent down-regulated functions and pathways, and the enrichment of gene ontology terms was tested using Fisher’s exact test (GeneSCF v1.1-p2). (**E** and **J**) Upstream regulation analysis. Color legend: light red, buffer; light blue, Nest1; dark red, ZIKV buffer; dark blue, ZIKV Nest1.

To deeply characterize the immune properties driven by Nest1, we performed a transcriptomic analysis of the human skin samples up to 4 days after inoculation with Nest1. Principal components analysis (PCA) revealed that Nest1-incubated skin explants (light blue) formed a separate cluster from the explants used as a control (buffer; light red), being more evident at day 1 after inoculation (Fig. 6A and fig. S6). Forty-eight genes were differentially expressed in the explants inoculated with Nest1 compared with the control (buffer) at day 1 after inoculation (Fig. 6B, right). To investigate the mechanisms by which Nest1 alters the host immune responses, we next defined the effect that Nest1 induces on cutaneous immune responses. The differential analysis comparing the control versus Nest1 identified the down-regulation of 26 genes and the up-regulation of 22 genes (Fig. 6C). Gene ontology functional analysis showed that down-regulated genes were clustered in the antiviral and antimicrobial response, innate immune response, or inhibition of RNA viruses, among other immune-related functions (Fig. 6D, top). Pathway analysis showed that Nest1 can inhibit the macrophage classical activation signaling pathway (*P* = 0.0008), hypercytokynemia-hyperche-mokynemia (*P* = 2.18776 × 10^−8^), role of RNA-activated protein kinase (PKR) in IFN induction and antiviral response (*P* = 0.0141), and IFN signaling pathways (*P* = 7.76247 × 10^−8^) (Fig. 6D, bottom). These effects were more evident at days 1 and 2 after inoculation based on the PCAs that were still observed even 3 days after inoculation (fig. S6). Upstream regulation analysis showed that IFN-stimulated genes, including IFIT1, IFIT3, IFI6, IFI44, IFI44L, or USP18; proinflammatory chemokines like CXCL9, CXCL10, or CXCL11; and antiviral genes such as DDX60, RSAD2 (viperin), PARP9, STAT1, RIGI, MX1, IFIH1, or EIF2AK2, were repressed (Fig. 6E). In addition, functions related to virus replication and infection were up-regulated (Fig. 6D, top). These findings support our results in which Nest1 exerts inhibitory effects over the host immune responses and facilitates virus dissemination.

To investigate the mechanisms by which Nest1 enhances flavivirus infection, a parallel analysis was performed including ZIKV in the presence (dark blue) and absence (dark red) of Nest1. A similar effect was also observed when we compared samples infected with ZIKV (ZIKV buffer; dark red) and samples infected with ZIKV and inoculated with Nest1 (ZIKV Nest1; dark blue) (Fig. 6 and fig. S7). PCA showed two well-defined clusters from the explants used as a control (ZIKV buffer) and those treated with Nest1 (ZIKV Nest1) (Fig. 6F). A total of 360 genes were differentially expressed at day 1, where 219 genes were down-regulated and 141 genes were up-regulated (Fig. 6, G and H). Genes involved in the activation of macrophages and inflammatory responses were repressed in the presence of Nest1, whereas genes involved in viral infection or infection by *Flaviviridae* were enhanced (Fig. 6I, top). Pathway analysis showed that Nest1 inhibits macrophage and proinflammatory cytokine-related pathways such as LXR/RXR activation (*P* = 3.31131 × 10^−5^), hypoxia-inducible factor 1α (HIF1α) signaling (*P* = 0.019054607), hypercytokynemia-hyperchemokynemia (*P* = 0.025703958), and IL-12 signaling pathways (*P* = 0.012302688) also when ZIKV is present (Fig. 6I, bottom). In this case, the upstream regulation analysis revealed that proinflammatory chemokines and IFN-stimulated and antiviral genes like CXCL11, CX3CL1, IFIT1, IFIT2, IFIT3, or RSAD2 were also found among the down-regulated genes, whereas proviral genes like CCR5 or MSR1 were up-regulated (Fig. 6J). These results indicate that the inhibitory effects of Nest1 over IFN-stimulated genes, as well as proinflammatory and antiviral components, are also present during ZIKV infection.

## DISCUSSION

Salivary proteins from hematophagous arthropods contain a complex cocktail of antihemostatic and immunomodulatory molecules to enable blood feeding. This can alter the local skin environment and affect the transmission of arthropod-borne pathogens (15, 19). Although the proviral effect of some salivary factors in arbovirus transmission has been elucidated in the past years, less is known about their interactions with the host cellular machinery. In this work, we demonstrate that the *Aedes* salivary protein Nest1 strongly interacts with CD47, a molecule expressed on many cell types, which controls multiple immunoregulatory functions. In addition, we used in vitro human skin explants, which are a crucial step in understanding infection, and showed that Nest1 increased the viral burden using ZIKV as a model of arbovirus infection. Last, as shown by the in vitro studies and transcriptomic analysis, we delineate how this protein can inhibit immune functions such as phagocytosis or cell migration, down-regulate several immune pathways, and up-regulate others involved in virus replication.

Nest1 is predominantly produced in the salivary glands of female *A. aegypti*, suggesting an important role in blood feeding behavior. In addition, studies have shown that a Nest1 homolog in *A. albopictus* modulates the length of intradermal probing before blood engorgement, indicating an additional role in the feeding process (51). We have also identified several salivary antigens in other hematophagous mosquito species (*A. albopictus*, *Culex* species, and *A. subalbatus*) that share homology with *A. aegypti* Nest1 and detected one homolog (fig. S1) in *C. quinquefasciatus*. This suggests that Nest1 may be functional in other mosquito genera and have a role for the transmission of other pathogens, such as West Nile or chikungunya viruses. The role of Nest1 in facilitating pathogen to human transmission has not been directly demonstrated, and murine models often do not recapitulate human disease. Human skin explants represent the closest way to mimic human infection without directly infecting volunteers, enabling us to specifically administer Nest1 into the tissue. Here, we show that the inoculation of Nest1 increases the ZIKV burden in human skin explants. This is consistent with studies showing that blocking Nest1 alters the microenvironment at the mosquito bite site and reduces ZIKV infection in immune-deficient mice (20). This model bypasses the limitations of in vitro cell culture assays and immunocompromised mouse models, which hinder the experimental study of many aspects of the human immune responses against arbovirus infections. These explant studies also strongly support that Nest1 plays an important role in the human response to mosquito salivary factors. In addition, multiple cell types can be infected by arboviruses, like skin muscle cells, which appear to be an early target of arbovirus infection (52), dermal fibroblasts, epidermal keratinocytes, immature dendritic cells, and dermal macrophages (15, 52–54). Further elucidation of the influence of Nest1 on infection in these cell types and the role of circulating cells among the different tissues like skin and lymph nodes will increase the knowledge about how Nest1 influences the vertebrate host response against arbovirus infection.

We then used an ultrahigh-throughput screening strategy, named REAP, to examine the human ligands that interact with Nest1. We show that Nest1 has a specific interaction with human CD47, a ubiquitously surface-expressed protein that regulates pleiotropic immune functions including leukocyte phagocytosis, recognition of “self,” immune cell homeostasis and mucosal repair, leukocyte transepithelial migration, or inflammation (31, 55–59). The binding affinity of Nest1 for CD47 was much stronger than that of SIRPA, a natural human ligand for CD47. Although we demonstrate that the *Aedes* salivary protein Nest1 strongly interacts with CD47, it is always possible that additional local host molecules could also be influenced directly or indirectly by Nest1. This suggests that mosquitoes may have evolved to secrete a salivary protein to actively target cellular activities modulated by CD47 that may be related to blood intake and inadvertently with viral progression. Nest1 inhibited phagocytosis by macrophages and the expression of proinflammatory cytokines, two processes regulated by CD47 that play roles in both viral clearance and the host immune response to mosquitoes. That this phagocytic inhibitory effect is abrogated when the macrophage is not in the presence of Nest1 suggests that Nest1 may exert its effect on CD47 expressed on the macrophage. This is supported by a study that showed that CD47 can positively regulate phagocytosis by its interaction with other integrins on the surface of the macrophage (27). In addition, our transcriptomic analysis revealed a strong inhibition of cytokines, chemokines, and IFN-stimulated genes, such as members of the CXCL and IFIT families—host factors shown to have a broad-spectrum activity against the replication, spread, and pathogenesis of a range of human viruses (60). This combination could result in the suppression of antimicrobial, antiviral, and immune pathways in the skin triggered by Nest1 on one side and enhancement in pathways related to viral infection and replication on the other. These effects triggered by Nest1 can even last for days, aiding the dissemination of the pathogen. A study has shown that the effect of mosquito bites can be notable even 48 hours after saliva delivery (61). Additional data (fig. S5) suggest that Nest1 may also inhibit human neutrophil chemotaxis, another immune activity also controlled by CD47 (34), and the mechanism may be multifactorial. We have unraveled a strong interaction between the human CD47 and Nest1 mosquito salivary protein, which alters some host immune functions. However, it is possible that Nest1 could also interact with other factors, regulating host mechanisms that are not considered in this work. In addition, new roles of CD47 are being identified. Different forms and ligands like SLAMF7 (SLAM family member 7) (62) are also being found, demonstrating the complexity of the pathways regulated by CD47.

Mosquito saliva influences viral transmission by altering different host pathways. We now show that the secreted *A. aegypti* salivary protein Nest1 interacts specifically and strongly with human CD47. This interaction modulates the local immune response and enhances viral infectivity in human skin explant studies. The observations described in this work open a panel of multiple questions: The importance of this interaction in diverse genera of mosquitoes and in the pathogenesis of different viruses remains to be uncovered, as does the importance of Nest1 in a variety of other CD47-mediated responses. Other local host molecules besides CD47 could also interact with Nest1, triggering effects that were not evaluated in this study. Understanding how cross-phyla signaling influences host responses suggests new ways to prevent vector-borne diseases and raises the possibility that Nest1 may be useful to treat human illnesses that are amenable to CD47-mediated intervention.

## MATERIALS AND METHODS

### Study design

The goal of this work was to study how a mosquito salivary antigen, Nest1, can interact with the host, affecting its immune system and inadvertently promoting arbovirus dissemination. A high-throughput screening based on yeast expressing the human exoproteome was performed, identifying CD47 as a potential target, which was validated by different biophysical and biochemical approaches. Immune functional assays related to CD47 were performed, and the biological effect during infection was also analyzed using ex vivo human skin samples.

### Mosquito rearing, dissection, infection, and blood feeding

*A. aegypti* (Orlando strain) mosquitoes were maintained on 10% sucrose feeders ad libitum, inside a metal mesh cage [12 inches by 12 inches by 12 inches (30.48 cm by 30.48 cm by 30.48 cm); BioQuip, catalog no. 1450B] at 28°C and ~80% humidity with a 14-hour:10-hour light:dark photoperiod. Egg masses were generated via blood meal feeding on naïve SV/E 129 mice (Charles River Laboratories). All mosquitoes were housed in a warm chamber in a space approved for BSL2 and ACL3 research. Mosquitoes were used in these experiments 2 to 14 days after emergence. For dissection, mosquitoes were immobilized in a cold tray, and salivary glands, midguts, and ovaries were collected as previously described (63) and placed in RLT lysis buffer for further RNA extraction or 1X phosphate-buffered saline (PBS) for further protein immunoblot analysis. For mosquito infection, the arthropods were injected with 100 plaque-forming units of ZIKV MEX2–81 (accession number KX446950; propagated in C6/36 insect cells) via intrathoracic injection using a Nanoinject II injector (Drummond Scientific, United States). For mosquito blood feeding, female *A. aegypti* were allowed to feed on ketamine-anesthetized wild-type mice with SV/E 129 for 20 min. For the mosquito odorant stimulation test, mosquitoes were placed into paper cups with a mesh on top. Cups were placed at the bottom of a cage containing three mice for 30 min. After 30 min, mosquitoes were ice-anesthetized, and salivary glands were dissected and placed in RLT buffer for further RNA extractions. Animals used for mosquito feeding were treated in accordance with guidelines from the Guide for the Care and Use of Laboratory Animals (National Institutes of Health). The animal experimental protocols were approved by the Institutional Animal Care and Use Committee (IACUC) at the Yale University School of Medicine (assurance number A3230–01). Every effort was made to minimize murine pain and distress. The mice were anesthetized with ketamine-xylazine for mosquito feeding experiments and euthanized as suggested by the Yale IACUC.

### Cells and virus culture

Stellar competent cells (*Escherichia coli* HST08 strain) were cultured at 37°C in LB medium, and ampicillin (1 mg/ml) was used to select transformed clones. *Saccharomyces cerevisiae* strain JAR300 was maintained in SDO-Ura medium (synthetic dropout medium; US Biological, no. D9535 prepared with 20 g/liter glucose, according to the manufacturer’s instructions). Expi293 cells (Thermo Fisher Scientific, no. A14527) were cultured in suspension at 37°C in 8% CO_2_ using Expi293 expression medium. Vero (CCL-81) and C6/36 (CRL-1660) cells were both obtained from the American Type Culture Collection (ATCC) and cultured in Dulbecco’s modified Eagle’s medium supplemented with 10% fetal bovine serum (FBS) and 1% penicillin-streptomycin or Leibovitz L-15 medium supplemented with 10% FBS and 1% penicillin-streptomycin at 37° and 30°C, respectively. BHK-21 (clone 15) cells were cultured in minimum essential medium supplemented with 10% FBS, 1% glutamine, 1% penicillin-streptomycin, and sodium bicarbonate at 37°C in 5% CO_2_. THP-1 (TIB-202) cells obtained from the ATCC and U937 cells (CRL-1593.2), kindly provided by K. Miller-Jensen in the Department of Biomedical Engineering at Yale University, were cultured at 37°C in 5% CO_2_ in RPMI medium supplemented with 10% FBS, 1% glutamine, and 1% penicillin-streptomycin. ZIKV Mex I44 and ZIKV MEX2–81 (accession number KX446950) were obtained from the World Reference Center for Emerging Viruses and Arboviruses at the University of Texas, Medical Branch (Galveston, TX). For mosquito infections, ZIKV MEX2–81 was successively passaged in C3/36 cells. For the human skin explant studies, ZIKV Mex I44 was cultured in Vero cells (ATCC CCL-81) for four passages and then passaged once in C3/36 cells as referenced (64, 65). ZIKV titrations were performed by plaque assay in BHK cells or focus-forming assay as previously described (66).

### Human primary cell lines and skin explants

Human whole white blood cells, RBCs, monocytes, and PMNs were collected and also cultured at 37°C in 5% CO_2_ in RPMI medium. Whole white blood cells, monocytes, and PMNs were isolated from whole blood obtained from healthy human volunteers, with approval from the University of Michigan and Yale University School of Medicine Institutional Review Board on human participants. Deidentified adult human skin was donated by fully informed patients, who provided written consent, undergoing reduction mammoplasty at the Upstate Medical University Hospital (Syracuse, NY). All skin specimens were collected within 2 hours after surgery.

### RNA extraction, cDNA synthesis, and qRT-PCR–based assays

All mosquito RNA extractions and RNA from peripheral human white blood cells were performed using RLT lysis buffer according to the manufacturer’s protocol (QIAGEN). The RNA was subsequently used for production of a cDNA pool with iSCRIPT (Bio-Rad). The qRT-PCR assays were done using the iTaq kit according to the manufacturer’s instructions (Bio-Rad). Viral RNA or *Aedes* gene expression was normalized to Rp49 expression. Human cytokine expression was normalized to human glyceraldehyde-3-phosphate dehydrogenase (GAPDH). RNA isolation and qRT-PCR in skin (*n* = 4) and skin culture media (*n* = 4) were harvested in 24-hour time intervals until the end point at 4 days after infection. Each skin sample was placed in TRIzol reagent and homogenized using two steel beads for 5 min at 30 Hz using a Qiagen TissueLyser II. Tissue RNA was isolated using a solid-phase extraction (Qiagen RNeasy Mini Kit), whereas skin culture medium samples were placed in TRIzol LS, allowing for RNA extraction using the QIAcube HT (Qiagen RNeasy 96 HT). RNA purity and concentration were determined using a DeNovix DS-11+ spectrophotometer. qRT-PCR was performed using the iTaq Universal Probe One-Step Kit (Bio-Rad) combined with a modified primer probe combination previously identified (67). Probe ZIKV 1107-Fam was modified from its original formulation to use an Iowa Black (FQ) dark quencher (IDT, Coralville, Iowa). Absolute quantification of viral loads was calculated using a standard curve. Focus-forming units (FFUs) were normalized to μg of RNA by isolating RNA from a known high-titer ZIKV stock and generating a standard curve using 10-fold serial dilutions. The standard curve was generated by plotting log_10_ FFUs (from the known titer virus) as a function of cycle threshold (Ct) value. The generated standard curve (*y* = −3 − 223*x* + 4 − 0.541, *R*^2^ = 0.9943) had a calculated amplification efficiency of 104%. Sample Ct values were applied to the standard curve to calculate the log_10_ FFUs, which were normalized to μg of RNA to account for variations in total RNA mass from different skin samples. The numbers reported were log_10_ FFU normalized to μg of RNA. Oligos for the qRT-PCR reactions are shown in table S1.

### Nest1 recombinant protein expression, protein immunoblot, and coimmunoprecipitation

*A. aegypti* Nest1 (amino acids 22 to 316) was cloned using the In-Fusion HD Cloning Kit (Takara) into pEZT-Dlux, a modified pEZT-BM vector that includes the insertion of an H7 leader sequence upstream followed by an AviTag (Avidity), an HRV 3C site, a protein C epitope, and an 8X His tag downstream, and transformed into Stellar competent cells (Takara Bio) for plasmid amplification. Expi293 cells were transfected with the Nest1-pEZT construct using the ExpiFectamine 293 Transfection Kit (Thermo Fisher Scientific, no. A14524). Recombinant protein was purified from clarified supernatants using a Ni–nitrilotriacetic acid (NTA) agarose affinity column (Qiagen) and concentrated and desalted into PBS by spin filtration using ultra 10-kDa centrifugal filters (Amicon, Millipore). rNest1 protein expression was tested by protein immunoblot using rabbit anti-Nest1 1:1000 (Cocalico Biologicals), goat anti-rabbit horseradish peroxidase (HRP) antibody (Thermo Fisher Scientific), and anti-his–HRP antibodies (Abcam) (see all unmodified protein immunoblots in fig. S8). Protein purity was verified by SDS–polyacrylamide gel electrophoresis. Protein concentration was measured by BCA assay (Thermo Fisher Scientific) at 562 nm. For in vitro assays, Nest1 protein was purified under endotoxin-free conditions and collected in 150 mM NaCl and 10 mM Hepes buffer. *C. quinquefasciatus* 34k2 protein was generated/tested and quantified following the protocols mentioned above. Native mosquito Nest1 or 34k2 was detected in mosquito tissues by protein immunoblot using rabbit anti-Nest1 antibodies. Human CD47 (19 to 135) recombinant protein was also expressed using this expression system tagged with 8X His or with human immunoglobulin G1 Fc (N297A). Coimmunoprecipitation assays were performed using protein A/G magnetic beads (Pierce). Briefly, 5 μg of recombinant human CD47-Fc protein (Sino Biological) was coupled to 50 μl of A/G magnetic beads and incubated with 1 μg of Nest1 or *A. aegypti* AesNAP protein (negative control). Uncoupled beads were left untreated and incubated with Nest1 as an additional negative control. Aliquots from input, flow-through, wash, and immunoprecipitation were tested for the presence of Nest1 by protein immunoblot using anti-his–HRP antibody.

### Yeast library screening

Details of yeast library construction and screenings have been previously described (43). Briefly, a library of barcoded plasmids containing the ectodomain and extracellular sequences of 2688 human proteins was expressed in *S. cerevisiae* strain JAR300 and maintained in SDO-Ura medium (US Biological, no. D9535 prepared with 20 g/liter glucose, according to the manufacturer’s instructions). The expression of recombinant proteins over the yeast surface was induced by culturing the yeast in 90% galactose–10% glucose medium for 24 hours at 30°C. Induced yeast cells (1 × 10^7^) were harvested in a sterile 96-well v-bottom microtiter plate. Cells were resuspended in 100 μl of PBE [PBS + 0.5% w/v bovine serum albumin (BSA) + 0.5 mM EDTA] with 10 μM biotinylated (BirA500 kit, Avidity) rNest1 protein and incubated for 1 hour at 4°C with shaking. Yeast cells were washed once with 200 μl of PBE, resuspended in 100 μl of PBE containing 1 μl of streptavidin microparticles (Spherotech, 0.29 μm, no. SVM-025–5H), and incubated for 1 hour at 4°C with shaking. Yeast cells were washed once with 200 μl of PBE, and bead-bound cells were selected by magnetic separation and subsequently expanded in 1 ml of SDO-Ura supplemented with chloramphenicol at 30°C. Linearized yeast-display vectors encoding the human proteins (pDD003 digested with Eco RI and Bam HI) were extracted from selected yeast cell libraries using Zymoprep-96 Yeast Cell Plasmid Miniprep kits or Zymoprep Yeast Cell Plasmid Miniprep II kits (Zymo Research) according to the standard manufacturer’s protocols. DNA was amplified with custom primers and sequenced using an Illumina MiSeq and Illumina v2 MiSeq Reagent Kits according to the standard manufacturer’s protocols. Barcode counts were extracted from raw NGS data using Python. All enrichment calculations were performed using edgeR (68). The score for each gene was defined as the overall enrichment for that gene (relative to the unselected library) multiplied by the percentage of barcodes associated with the gene that enriched (defined as logFC > 0). A negative log fold change (logFC) means a protein was depleted; therefore, for simplicity, all negative values were set to zero.

### Flow cytometry for Nest1-CD47 interaction studies

Six different constructs of Nest1 were generated (full-length and amino acids 22 to 126, 22 to 153, 22 to 216, 126 to 316, and 153 to 316) and cloned into the pDD003 yeast-display vector for further surface expression in *S. cerevisiae* strain JAR300, as described above. Yeast cells were then incubated for 1 hour at 4°C with 1 μM recombinant CD47-Fc protein previously expressed in Expi293 cells and labeled with anti-Fc–phycoerythrin (PE) antibody (BioLegend) for flow cytometry analysis. Anti-FLAG–PE antibody (BioLegend) was used as a control for Nest1 expression in yeast and run through an SA3800 Spectral Analyzer (Sony Biotechnology). The data were analyzed by FlowJo.

### Analytical SEC

His-tagged CD47 (19 to 135) and Nest1 (22 to 316) proteins were used for analytical SEC. The Nest1-CD47 complex was formed by incubating 1.2× molar excess of CD47 with Nest1 at room temperature (RT) for 45 min before injecting onto an ENrich SEC 70 column (Bio-Rad). Equivalent molar amounts of CD47 and Nest1 were injected separately to compare with the elution volume of the complex.

### Surface plasmon resonance

Affinity measurements were performed on a BIAcore T200 instrument using a Biotin CAPture kit (Cytiva). CD47-Fc expression supernatant was subjected to protein A agarose purification (GoldBio) and further purified by SEC. CD47-Fc was biotinylated using *N*-hydroxysuccinimide (NHS)–biotin (Thermo Fisher Scientific) using a 10-fold molar excess of NHS-biotin over protein. Excess biotin was removed using a PD-10 column (Cytiva). Nest1-his was expressed and purified by Ni-NTA chromatography and SEC. CD47 was immobilized to the chip surface at low density (RUmax < 100 response units), and serial dilutions of Nest1 were exposed to the surface for 120 s. Dissociation was then tracked for 60 s. To determine whether Nest1 shares the same binding site as SIRPα, we flowed twice 50 nM CV-1 over the chip to completely block CD47 followed by an injection of 100 nM Nest1. Experiments were performed in HBS-P+ buffer (Cytiva) at 25°C at a flow rate of 30 μl/min. Data analysis and determination of equilibrium and kinetic parameters were carried out with the Biacore T200 evaluation software assuming a 1:1 Langmuir binding model. Binding curves were exported and plotted using the Prism software (GraphPad).

### Hydrogen-deuterium exchange mass spectrometry

HDX-MS experiments were performed by Creative Proteomics (Shirley, NY) following a previously published protocol with minimal modifications (69). Details of reaction conditions and all data are presented in HDX tables. Three conditions were tested and compared: (i) CD47, (ii) Nest1, and (iii) CD47-Nest1 complex. HDX reactions were done in 50-μl volumes with a final protein concentration of 3.8 μM. Briefly, 188 pmol of protein was preincubated in 5.8-μl final volume for 5 min at 22°C before initiating deuteration reaction.

Deuterium exchange reaction was initiated by adding 45 μl of D_2_O exchange buffer [20 mM Tris (pH 7.5), 137 mM NaCl, and 3 mM KCl] to the protein mixture. Reactions were carried out at RT for three incubation times (3, 30, and 300 s) and terminated by the sequential addition of 20 μl of ice-cold quench buffer 1 [4 M guanidine HCl, 1 M NaCl, 100 mM NaH_2_PO_4_ (pH 2.4), 1% formic acid (FA), and 100 mM TCEP]. Samples were immediately frozen in liquid nitrogen and stored at −80°C for up to 2 weeks. All experiments were repeated in triplicate. To quantify deuterium uptake into the protein, we thawed samples and injected them into an ultra-performance liquid chromatography (UPLC) system immersed in ice with 0.1% FA as the liquid phase. The protein was digested via two immobilized pepsin columns (Thermo Fisher Scientific, no. 23131), and peptides were collected onto a VanGuard precolumn trap (Waters). The trap was subsequently eluted, and peptides were separated with a C18, 300-Å, 1.7-μm particle size Fortis Bio 100 × 2.1 mm column over a gradient of 8 to 30% buffer C over 20 min at 150 μl/min (buffer B, 0.1% FA; buffer C, 100% acetonitrile). Mass spectra were acquired on an Orbitrap Velos Pro (Thermo Fisher Scientific), for ions from 400 to 2200 *m*/*z* (mass/charge ratio) using an electrospray ionization source operated at 300°C and 5 kV of ion spray voltage. Peptides were identified by data-dependent acquisition of a nondeuterated sample after tandem mass spectrometry, and data were analyzed by Mascot. All peptides analyzed are shown in HDX tables (fig. S3 and data files S1 and S2).

Deuterium incorporation levels were quantified using the HD examiner software (Sierra Analytics), and the quality of every peptide was checked manually. Results are presented as the percentage of maximal deuteration compared with theoretical maximal deuteration. Changes in deuteration level between two states were considered significant if >5% and >0.5 Da and *P* < 0.05 (unpaired *t* test).

### Human whole white blood cell, monocyte, and PMN isolation from peripheral blood

Peripheral blood samples were collected into precoated heparin tubes and diluted in PBS. For whole blood cell preparations, samples were treated with ACK lysis buffer for 5 min on ice for erythrocyte lysis. For monocyte and PMN isolation, samples were submitted to a Ficoll-Paque Plus (Sigma-Aldrich, St. Louis, MO, United States) density gradient centrifugation protocol at 450*g* for 30 min at RT. Peripheral blood mononuclear cells (buffy coat) were then collected and treated with ACK lysis buffer for 5 min on ice to lyse the remaining erythrocytes. The cells were counted in a hemocytometer, and monocytes were sorted by negative selection using the Classical Monocyte Isolation Kit (Miltenyi Biotec). Cells were then differentiated into macrophages for 5 days after adding human macrophage colony-stimulating factor (30 ng/ml; Miltenyi Biotec). PMNs were isolated using a previously described Polymorphprep (Axis Shield) density gradient centrifugation technique (70). PMNs were resuspended in Hanks’ balanced salt solution (HBSS) with 10 mM Hepes (pH 7.4) and without Ca^2+^ or Mg^2+^ at a concentration of 5 × 10^7^ cells/ml. Neutrophils isolated in this way were 97% pure and >95% viable and were used for transmigration within 2 hours of isolation. Whole white blood cells, monocytes, and PMNs were isolated from whole blood obtained from healthy human volunteers, with approval from the University of Michigan and Yale University School of Medicine Institutional Review Board on human participants.

### Immunofluorescence-based confocal imaging

Blood-derived human macrophages were grown on glass coverslips in a M12-well plate (1 million cells per well). Cells were washed twice with PBS, fixed with 4% paraformaldehyde in PBS for 20 min at RT, and permeabilized with PBS–0.1% Triton X-100 for 10 min at RT. Cells were blocked for 1 hour with PBS containing 5% BSA and incubated with primary polyclonal rabbit anti-huCD47 antibodies (Invitrogen) for 2 hours at RT. After three washes in PBS, the cells were incubated with Alexa Fluor 488–conjugated secondary antibodies (Life Technologies). After three final washes with PBS, cell actin was labeled with phalloidin CF 647 conjugate (Biotium) for 10 min at RT, and the coverslips were mounted on slides with prolong gold antifade reagent with 4′,6-diamidino-2-phenylindole (Invitrogen) for nuclei labeling. For suspension cells like THP-1 and U937, the same protocol was followed in 1.5-ml tubes, 5 million cells per tube, and the cells were deposited on glass coverslips at the end of the staining process. Cell imaging was performed using a Zeiss LSM 880 Airyscan confocal microscope (Carl Zeiss, Jena, Germany) with a 63× immersion oil objective at the Confocal Microscopy Core Facility of the Yale University School of Medicine.

### Phagocytosis assays

THP-1 cells, U937 cells, or blood-derived human macrophages (4 × 10^5^) were plated into temperature-responsive Nunc UpCell 12 multidishes (Thermo Fisher Scientific) overnight. Human RBCs (1 × 10^6^) were used as target cells for these experiments and labeled with CFSE, following the manufacturer’s instructions (CellTrace, Invitrogen). Cells were incubated for 4 hours at 37°C in the presence or absence of Nest1 protein and anti-human GlycA (BioxCell InVivoMAb, 6A7M) antibodies as opsonin at different concentrations. For preincubation assays, RBCs were pretreated with Nest1 protein and anti-human GlycA antibodies for 1 hour at 37°C and then washed. Pretreated cells were cocultured with macrophage cells for 4 hours at 37°C. After incubation, phagocytosis was analyzed by flow cytometry. Macrophages were identified with APC anti-human CD14 antibodies (BioLegend). Phagocytosis was quantified as the percentage of CD14^+^ cells that engulfed CFSE^+^/RBCs per total CD14^+^ population.

### PMN migration and CD11b activation and expression assays

Chemotaxis in PMNs was evaluated using collagen-coated (10 μg/cm^2^) 0.33-cm^2^ polycarbonate Transwells (3-μm pore size; Costar Corp., Cambridge, MA). Migration of PMNs (1 × 10^6^) added to the upper chamber of Transwell inserts was induced by a chemotactic gradient of 100 nM *N*-formylmethionine-leucyl-phenylalanine (fMLF) for 1 hour at 37°C. PMNs were pretreated with increasing concentrations of Nest1. Transmigrated PMNs into the bottom chamber were quantified by colorimetric enzyme activity assay specific for the PMN azurophilic marker myeloperoxidase. Activation of CD11b on PMNs was assessed using a monoclonal antibody that recognizes an epitope that is only displayed on activated integrins that are in a high-affinity binding conformation (CBRM1/5 clone; eBioscience, no. 17–0113-41) (71). Active CD11b on the plasma membrane was measured by flow cytometry at different time points after Nest1 treatment. Total CD11b expression (ICRF44 clone; BD Biosciences, no. 561687) in Nest1-treated cells stimulated with 100 nM fMLF was also measured and analyzed in this manner.

### Human whole white blood cell incubation with Nest1 for cytokine expression

Inflammatory response evaluation after Nest1 treatment was performed in white blood cells from four healthy donors. One million white blood cells per well (in triplicate) were preincubated in 96-well U-bottom plates (Falcon) for 1 hour with serum-free Iscove’s modified Dulbecco’s medium (IMDM) in the presence of Nest1 (2.9 μΜ) or buffer (negative control) and incubated for 24 hours for a final concentration of 1.45 μΜ in IMDM and 2% FBS. After incubation, cells were spun down for 5 min at 500*g* and lysed in RLT buffer for further RNA extraction.

### Preparation and inoculation of the human skin

Deidentified adult human skin was donated by fully informed patients, who provided written consent, undergoing reduction mammoplasty at the Upstate Medical University Hospital (Syracuse, NY). All skin specimens were collected within 2 hours after surgery. The skin was prepared as previously described (72, 73). Briefly, the skin was grossly dissected of underlying adipose, washed with PBS, cut into 1-cm^2^ sections, and then placed in Netwells (Corning) with a mesh size of 15 mm by 500 μm to be suspended at the air-liquid interface. The skin was incubated in RPMI 1640 medium supplemented with 10% FBS, 1% penicillin-streptomycin, and amphotericin B (0.25 μg/ml). The skin was intradermally inoculated using 28-gauge insulin syringes with 25-μl (±2 μl) injections adjusted to deliver 10^3^ FFUs of ZIKV. Experimental and control groups included Nest1 only, ZIKV and Nest1, ZIKV and buffer, and buffer only. rNest1 was diluted to deliver 3 μg to each skin sample. All experiments involving deidentified human specimens were conducted in a biological safety level 2 laboratory in accordance with a protocol approved by the SUNY Upstate Medical University (Syracuse, NY) Institutional Review Board.

### RNA sequencing analysis of Nest1 cutaneous transcriptional signatures during ZIKV replication

The RNA quality check, sequencing, and bioinformatics analysis were performed at Azenta Life Sciences (South Plainfield). RNA samples were quantified using a Qubit 2.0 Fluorometer (Life Technologies, Carlsbad, CA, United States), and RNA integrity was checked using an Agilent TapeStation 4200 (Agilent Technologies, Palo Alto, CA, United States). RNA sequencing libraries were prepared using the NEBNext Ultra II RNA Library Prep Kit for Illumina using the manufacturer’s instructions (NEB, Ipswich, MA, United States). Briefly, mRNAs were first enriched with oligo(dT) beads. Enriched mRNAs were fragmented for 15 min at 94°C. First-strand and second-strand cDNAs were subsequently synthesized. cDNA fragments were end-repaired and adenylated at the 3′ ends, and universal adapters were ligated to cDNA fragments, followed by index addition and library enrichment by limited-cycle PCR. The sequencing libraries were validated on the Agilent TapeStation (Agilent Technologies, Palo Alto, CA, United States) and quantified using the Qubit 2.0 Fluorometer (Invitrogen, Carlsbad, CA) and quantitative PCR (KAPA Biosystems, Wilmington, MA, United States). The sequencing libraries were clustered on flow cells. After clustering, the flow cells were loaded onto the Illumina HiSeq instrument (4000 or equivalent) according to the manufacturer’s instructions. The samples were sequenced using a 2×150–base pair paired-end configuration. Image analysis and base calling were conducted by the HiSeq Control Software. Raw sequence data (.bcl files) generated from Illumina HiSeq were converted into fastq files and demultiplexed using Illumina’s bcl2fastq 2.17 software. One mismatch was allowed for index sequence identification. After investigating the quality of the raw data, sequence read was be trimmed to remove possible adapter sequences and nucleotides with poor quality using Trimmomatic v.0.36. The trimmed reads were mapped to the *Homo sapiens* reference genome (GRCh38) available on ENSEMBL using the STAR aligner v.2.5.2b.

### Differential gene expression and pathway analysis

After the extraction of gene hit counts, the gene hit counts table was used for downstream differential expression analysis. Using DESeq2 (74), a comparison of gene expression between the groups of samples was performed. Genes with adjusted *P* values of <0.05 and absolute log_2_ FCs of >1 were labeled as differentially expressed genes (DEGs) for each comparison. A gene ontology analysis was performed on the statistically significant set of genes by implementing the software GeneSCF (75). Qlucore Omics Explorer version 3.9 (Qlucore, Lund, Sweden) was used for data analysis and visualization including unsupervised PCA and hierarchical clustering heatmaps. The plot shows the samples in a two-dimensional (2D) plane spanned by their first two principal components. The top 500 genes, selected by highest row variance, were used to generate the plot. The global transcriptional change across the groups compared was visualized by a volcano plot. Last, a biclustering heatmap was used to visualize the expression profile of the DEGs sorted by their adjusted *P* value by plotting their log_2_ transformed expression values in samples.

Overrepresented pathways, biological functions, and upstream regulators were identified using Ingenuity Pathway Analysis (IPA; QIAGEN, Redwood City, CA) knowledgebase. For this, DEGs identifiers were mapped, and the overlap among these and canonical pathway, upstream regulators, and biological functions was calculated using one-tailed Fisher’s exact test (FDR *P* < 0.05). The interaction among upstream regulators, DEGs, and relevant functions was analyzed using IPA My Pathways tools.

### Quantification and statistical analysis

Flow cytometry, cytokine screening, qRT-PCR data, and gene ontology terms were analyzed with GraphPad’s Prism 9 software (GraphPad Software Inc., San Diego, CA) with the following statistical tests: Student’s *t* test, Mann-Whitney, or analysis of variance (ANOVA) and Fisher’s exact test. Values of *P < 0.05, **P < 0.01, and ***P < 0.001 were considered statistically significant.

## Supplementary Material

sm

data files

MDAR

## Data Availability

The raw fastq files and processed RNA sequencing data ready for exploration can be accessed and downloaded at the National Center for Biotechnology Information Gene Expression Omnibus Datasets repository (GSE239840). Other raw data used to generate figures can be found in data file S3. Data are publicly available as of the date of publication. All data were analyzed with standard programs and packages, as detailed in the text. Any additional information required to reanalyze the data reported here is available from the lead contact upon request. Materials used in this work, such as plasmids or proteins, are available upon request.
