# Supplementary material for: The human CD47 checkpoint is targeted by an immunosuppressive Aedes aegypti salivary factor to enhance arboviral skin infectivity
Source: Sci Immunol. Author manuscript; Available in PMC 2025 Mar 20. (PMC11924945; doi:10.1126/sciimmunol.adk9872)
Supplement: sm [file NIHMS2060361-supplement-sm.pdf]

Science Immunology
AAAS

Supplementary Materials for

The human CD47 checkpoint is targeted by an immunosuppressive *Aedes aegypti* salivary factor to enhance arboviral skin infectivity

Alejandro Marin-Lopez *et al.*

Corresponding author: Alejandro Marin-Lopez, alejandro.marinlopez@yale.edu;
Aaron M. Ring, aaronring@fredhutch.org; Erol Fikrig, erol.fikrig@yale.edu

Sci. Immunol. **9**, eadk9872 (2024)
DOI: 10.1126/sciimmunol.adk9872

The PDF file includes:

Figs. S1 to S8
Table S1

Other Supplementary Material for this manuscript includes the following:

Data files S1 to S3
MDAR Reproducibility Checklist

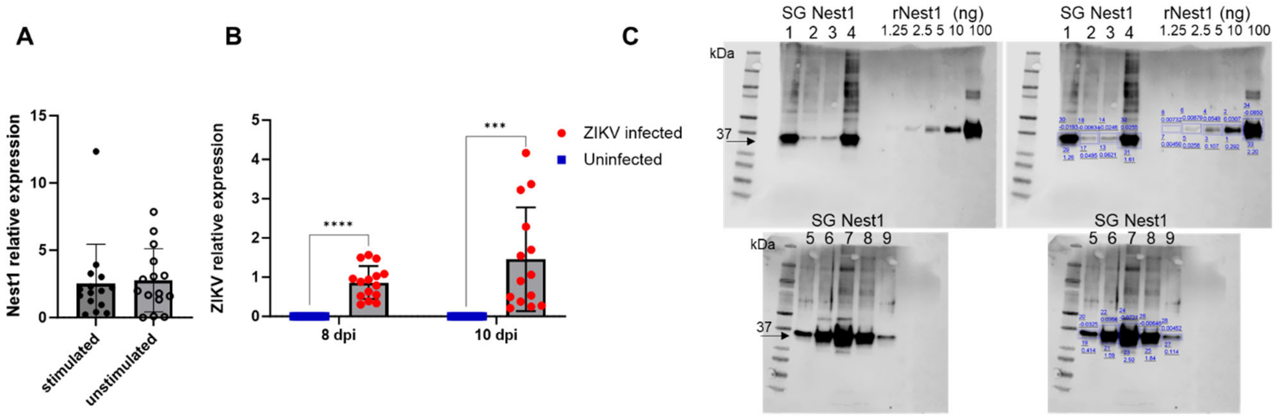

SG	band intensity	amount (ng)	standard	band intensity	amount (ng)
SG1	1.26	57.05096	1	0.0045	1.25
SG2	0.0495	2.1656795	2	0.0258	2.5
SG3	0.0621	2.7369761	3	0.107	5
SG4	1.61	72.92031	4	0.292	10
SG5	0.414	18.681296	5	2.2	100
SG6	1.59	71.97056			
SG7	2.5	113.2063			
SG8	1.84	83.29906			
SG9	0.114	5.087096			

	band intensity	amount (ng)	estimated (ng) to be secreted (half)	volume saliva secreted	estimated concentration
average SG	1.048844444	47.457582	23.72879098	2-5-250-1000nL	349-140-2.84-0.71µM

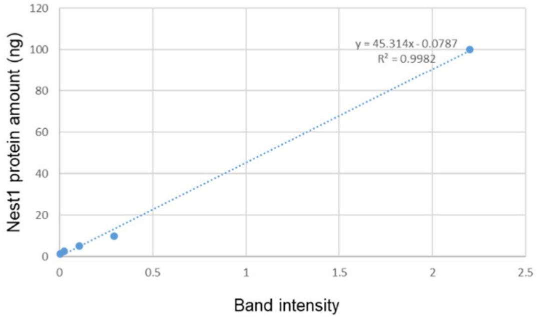

Supplementary figure 1. Characterization of Nest1 expression in *A. aegypti* salivary glands. (**A**) Relative expression of Nest1 levels in *A.aegypti* salivary glands after odorant stimulation. (**B**) Relative expression of ZIKV levels in the salivary gland of ZIKV infected and non-infected mosquitoes at day 8 and 10 post-infection. Rp49 was used as the housekeeping gene. (**C**) Amount (ng) and concentration (µM) of Nest1 in mosquito salivary glands. Protein immunoblot for native Nest1 expression in salivary glands (SG1-9) and recombinant Nest1 at known concentrations. Blot was probed with rabbit anti-Nest1 serum at a 1:1000 dilution. Relative band intensity quantification was measured by LI-COR software. Standard curve comparing the relative intensity of immunoblot bands for known amounts of recombinant Nest1 and the intensity of the specific bands corresponding to native Nest1 from salivary glands. Pearson correlation coefficient was calculated.

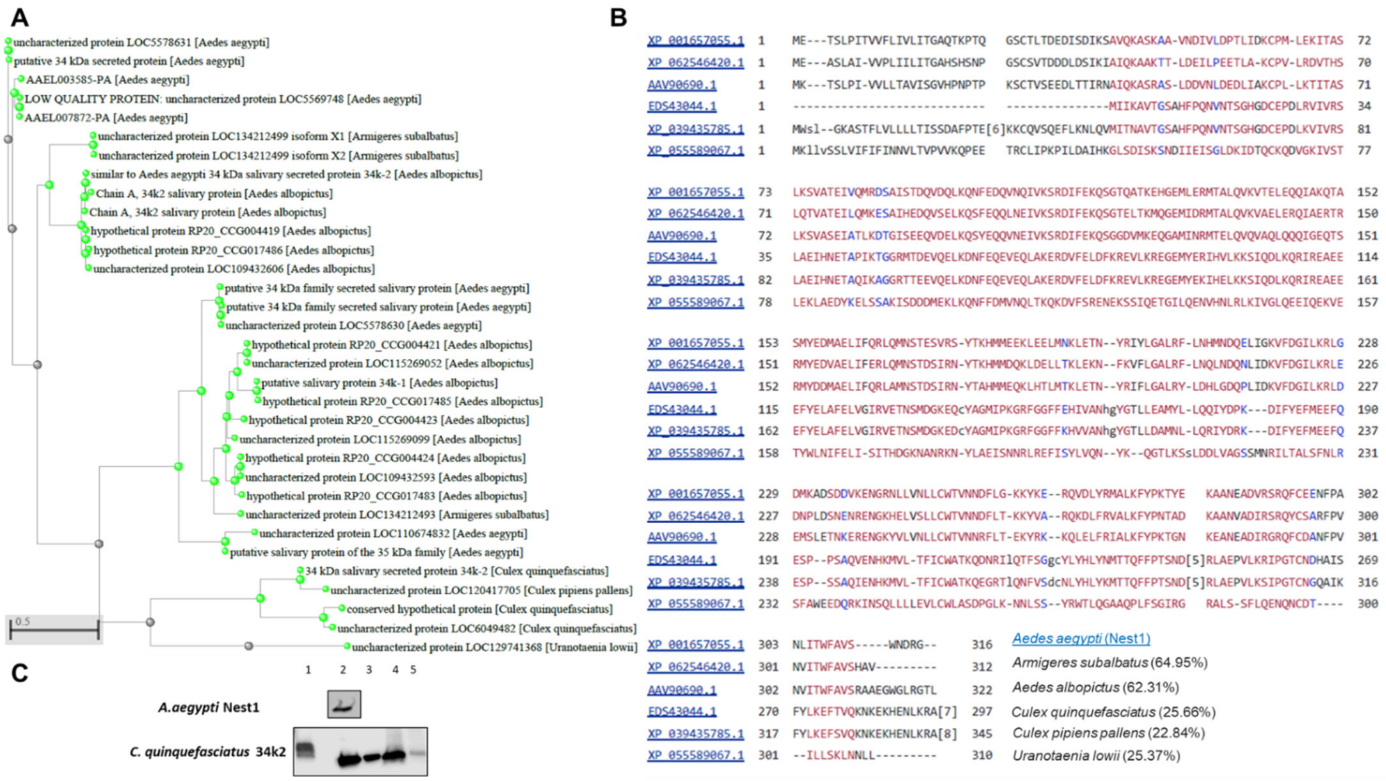

Supplementary figure 2. Homology studies of A. aegypti Nest1 amino acid sequence in other mosquito species. (**A**) Blast Tree View of *Aedes aegypti* Nest1 and homologs in other mosquito species. This tree was produced using BLAST pairwise alignments (NCBI). (**B**) Multiple alignment comparison produced by COLBALT (NCBI). Amino acid sequences of *Aedes aegpyti* Nest1 and five Nest1 homologs from other mosquito species: *Armigeres subalbatus*, *Aedes albopictus*, *Culex quinquefasciatus*, *Culex pipiens pallens* and *Uranotaenia lowii*) were compared, and percentage of identities relative to *A. aegypti* Nest1 are shown in parenthesis. (**C**) Cross reactivity of anti *A. aegypti* Nest1 antibodies against a Nest1 homolog in *C. quinquefasciatus* salivary glands. Lane 1 corresponds with the recombinant version of in *C. quinquefasciatus* 34k2 protein. Lanes 2-5 correspond with salivary gland extracts collected from *A.aegypti* (top) or in *C. quinquefasciatus* (bottom). Blot was probed with rabbit anti-Nest1 serum at a 1:1000 dilution.

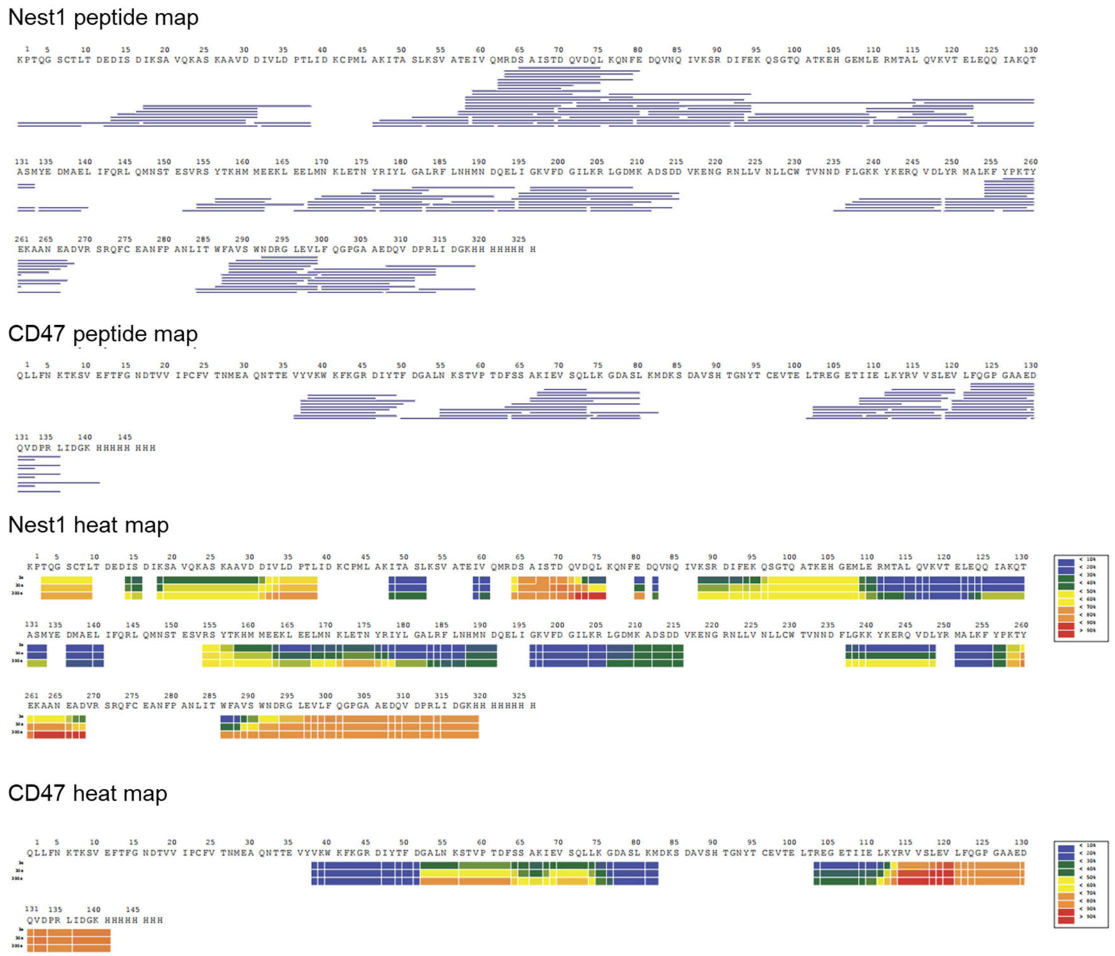

Supplementary figure 3. Nest1 and CD47 peptide and heat maps for the Hydrogen Deuterium Exchange Mass Spectrometry analysis. In peptide map, each line below the sequence represents a high-quality peptide that was analyzed by HDX-MS. In heat map, the line below the sequence of the protein is colored based on deuteration levels, according to the scale shown on the right. Percentage deuteration is shown as a theoretical maximum deuteration level. The three lines each represent percentage deuteration at different timepoints (3, 30, 300 seconds).

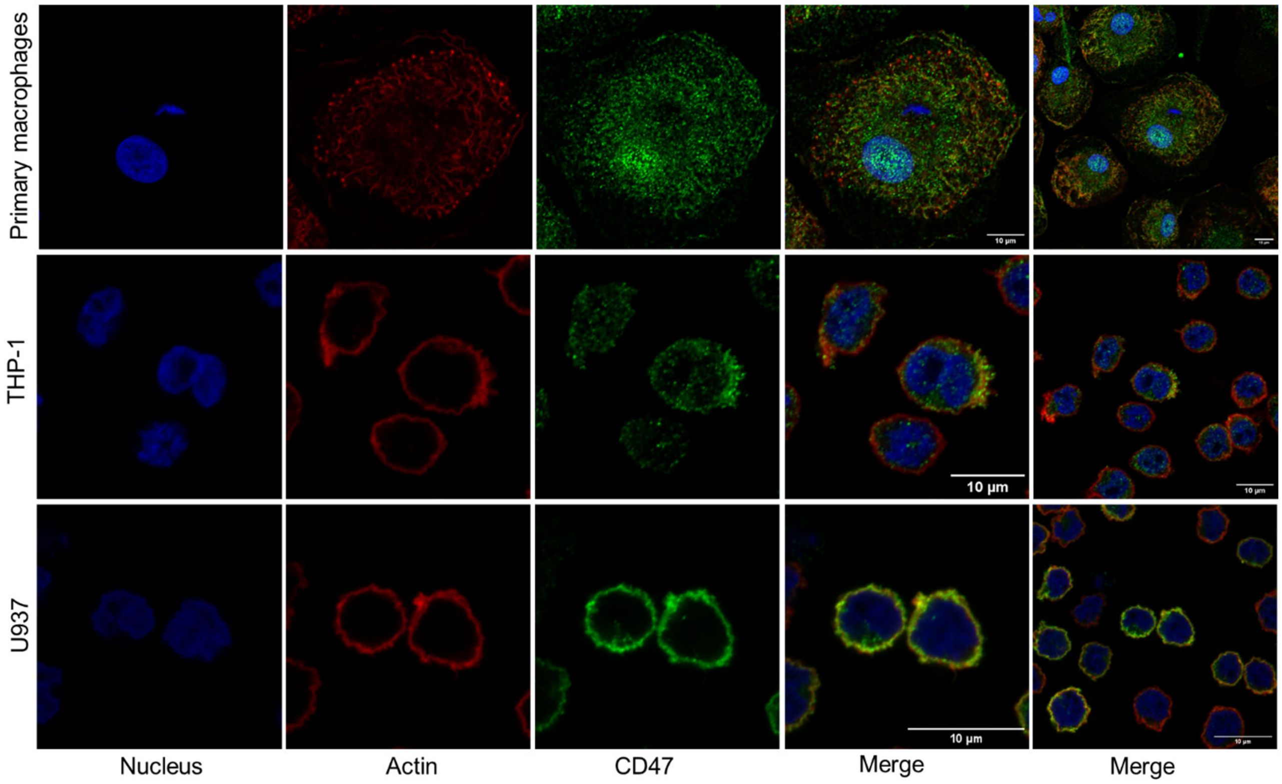

Supplementary figure 4. Confocal imaging analysis of CD47 surface expression in human primary macrophages, THP-1 and U937 cells. Cells were stained with a rabbit anti-human CD47 polyclonal antibody followed by anti-rabbit Alexa Fluor 488 (green). Actin (red) was stained using phalloidin and nucleus (blue) using DAPI. Images were captured by Zeiss LSM 880 Airyscan confocal microscopy. Images were analyzed using Fiji (Image J). Scale bars, 10 µm.

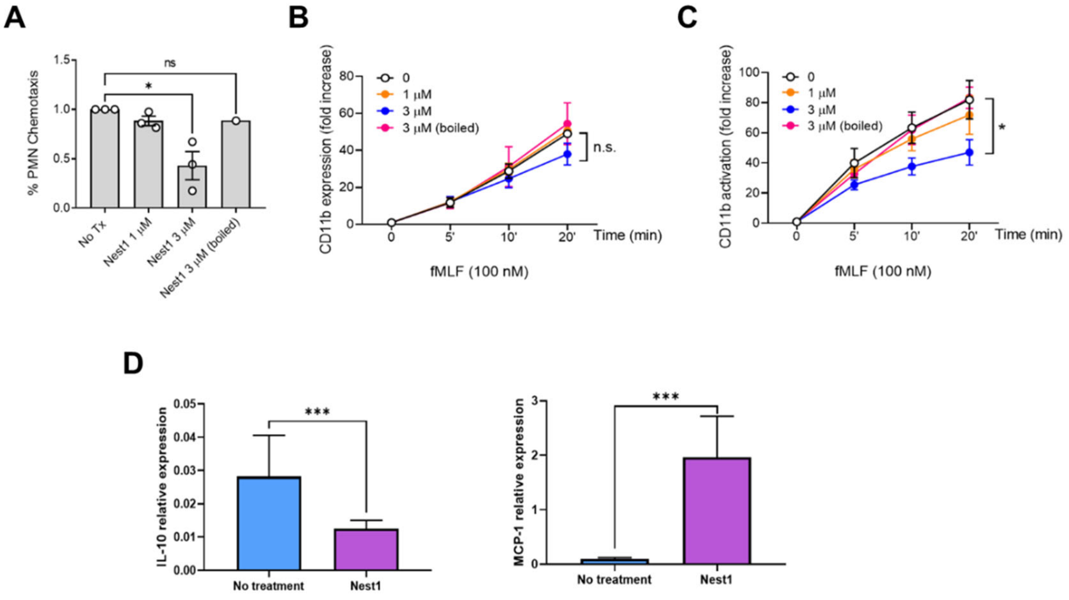

Supplementary figure 5. PMN migration assay and CD11b activation inhibited by Nest1 and IL-10 and MCP-1 gene expression in white blood cells after Nest1 treatment. (**A**) PMN chemotaxis assay induced by a chemotactic gradient of 100nM of N-Formylmethionine-leucyl-phenylalanine (fMLF). (**B**) CD11b surface expression in fMLF-induced PMN was determined by flow cytometry. (**C**) CD11b activation on fMLF-induced PMN was determined by using activation reporter mAbs, CBRM1/5. *p≤0.05, **p≤0.01 as determined by Two-way ANOVA analysis. Data are Means ± SEM of three independent experiments. (**D**) IL-10 and MCP-1 gene expression in white blood cells after Nest1 treatment. Expression was measured by qRT-PCR in non-treated and Nest1 treated human white blood cells, 24-hour post-stimulation. Cells were stimulated in triplicate and the measurements of four different donors were pooled. Human GAPDH was used as a housekeeping gene. These values represent the mean ± SEM of three replicates from a single experiment, including four different donors. Asterisks represent significant difference between samples, calculated by a student t-test ($p < 0.05$; *<0.05, **<0.01, ***<0.001).

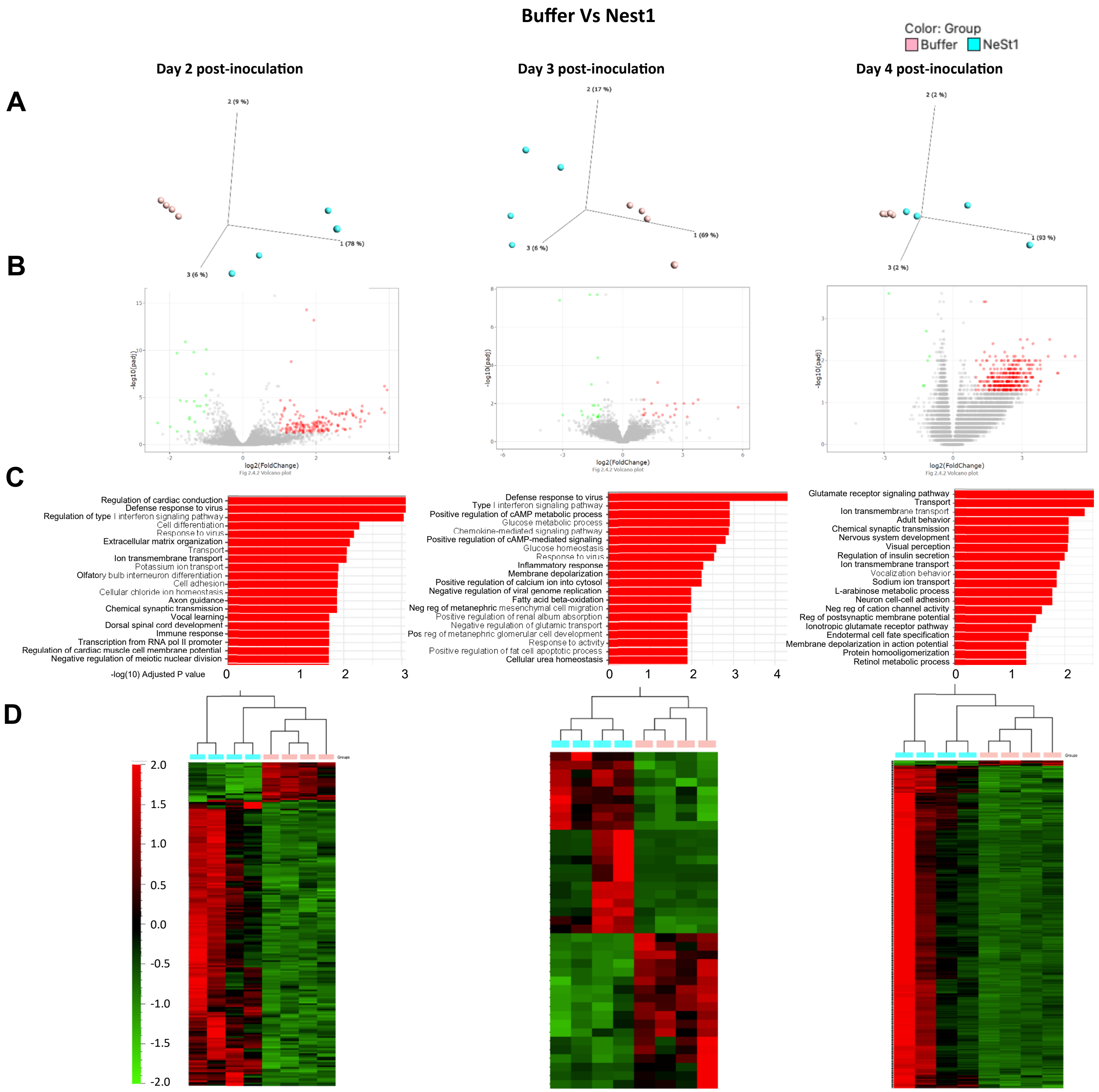

Supplementary figure 6. Transcriptomic analysis in human skin samples treated with Nest1. (A) Principal component assays and (B) volcano plots for the differentially expressed genes after Nest1 treatment, compared with control Buffer, at day two, three and four post-inoculation. The gene names can be found in Supplementary file 1. Principal component analysis reveals the similarity between samples based on

the distance matrix. Samples were projected to a 3D plane spanned by their first three principal components. The percentage of the total variance per direction is shown in the label. The global transcriptional change across the groups compared was visualized by volcano plots. Each data point in the scatter plot represents a gene. The log2 fold change of each gene is represented on the x-axis versus the -log10 of its adjusted p-value is on the y-axis. Genes with an adjusted p-value less than 0.05 and a log2 fold change greater than 1 are indicated by red dots. These represent up-regulated genes. Genes with an adjusted p-values of less than 0.05 and a log2 fold change less than 1 are indicated by green dots down-regulated genes. (C) Significant differentially expressed genes were clustered by their gene ontology. Red bars represent up-regulated functions. Green bars represent down-regulated functions. (D) A bi-clustering heatmap was used to visualize the expression profile of the differentially expressed genes sorted by their adjusted p-value by plotting their log2 transformed expression values in samples.

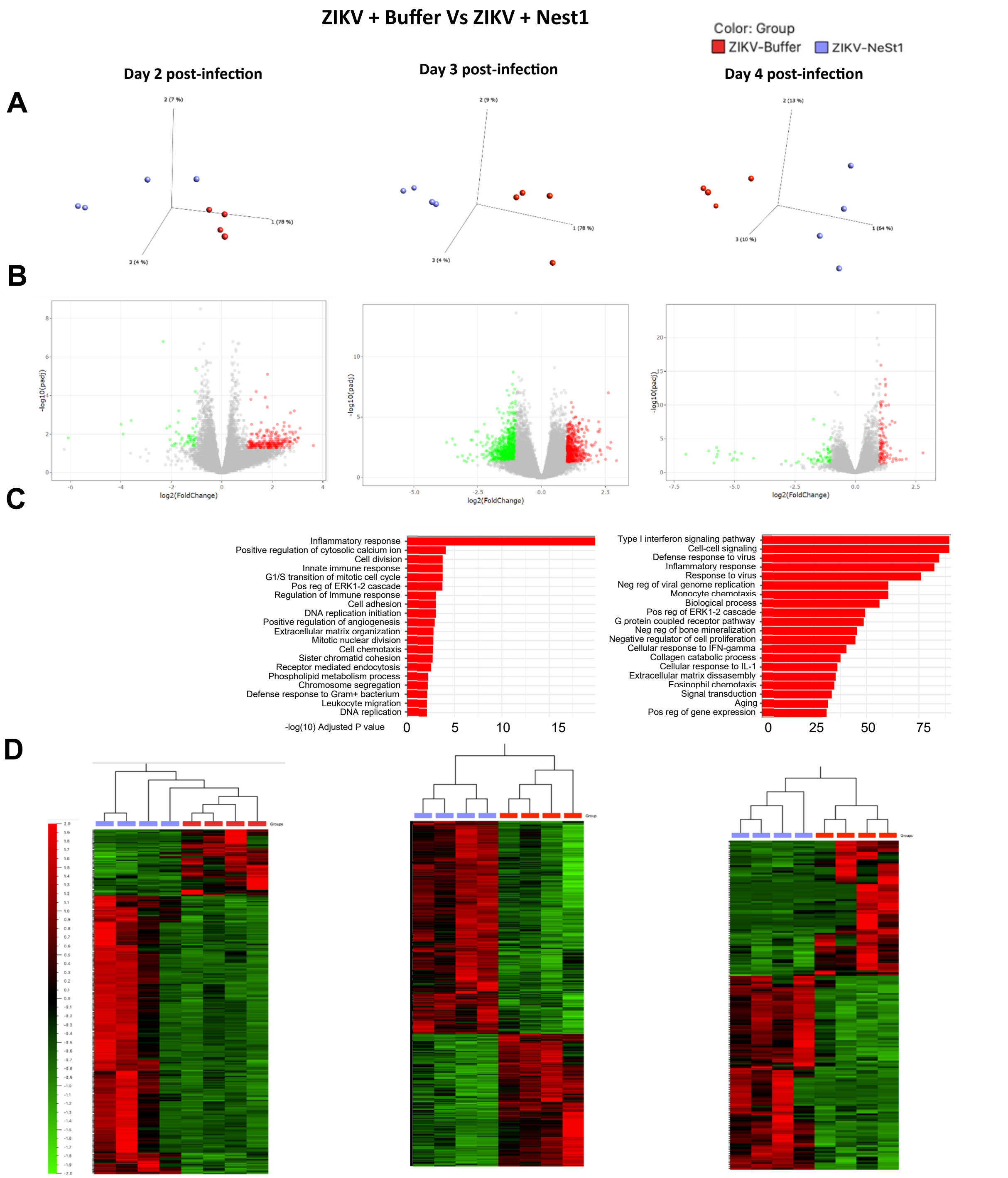

Supplementary figure 7. Transcriptomic analysis in human skin samples infected with ZIKV in the presence/absence of Nest1. (A) Principal component assays and (B) volcano plots for the differentially expressed genes after Nest1 treatment, compared with control Buffer, at day two, three and four post-infection. The gene names can be found in Supplementary file 1. Principal component analysis reveals the similarity between samples based on the distance matrix. Samples were projected to a 3D plane spanned by their first three principal components. The percentage of the total variance per direction is shown in the label. The global transcriptional change across the groups compared was visualized by

volcano plots. Each data point in the scatter plot represents a gene. The log2 fold change of each gene is represented on the x-axis versus the -log10 of its adjusted p-value is on the y-axis. Genes with an adjusted p-value less than 0.05 and a log2 fold change greater than 1 are indicated by red dots. These represent up-regulated genes. Genes with an adjusted p-values of less than 0.05 and a log2 fold change less than 1 are indicated by green dots down-regulated genes. (C) Significant differentially expressed genes were clustered by their gene ontology. Red bars represent up-regulated functions. Green bars represent down-regulated functions. (D) A bi-clustering heatmap was used to visualize the expression profile of the differentially expressed genes sorted by their adjusted p-value by plotting their log2 transformed expression values in samples

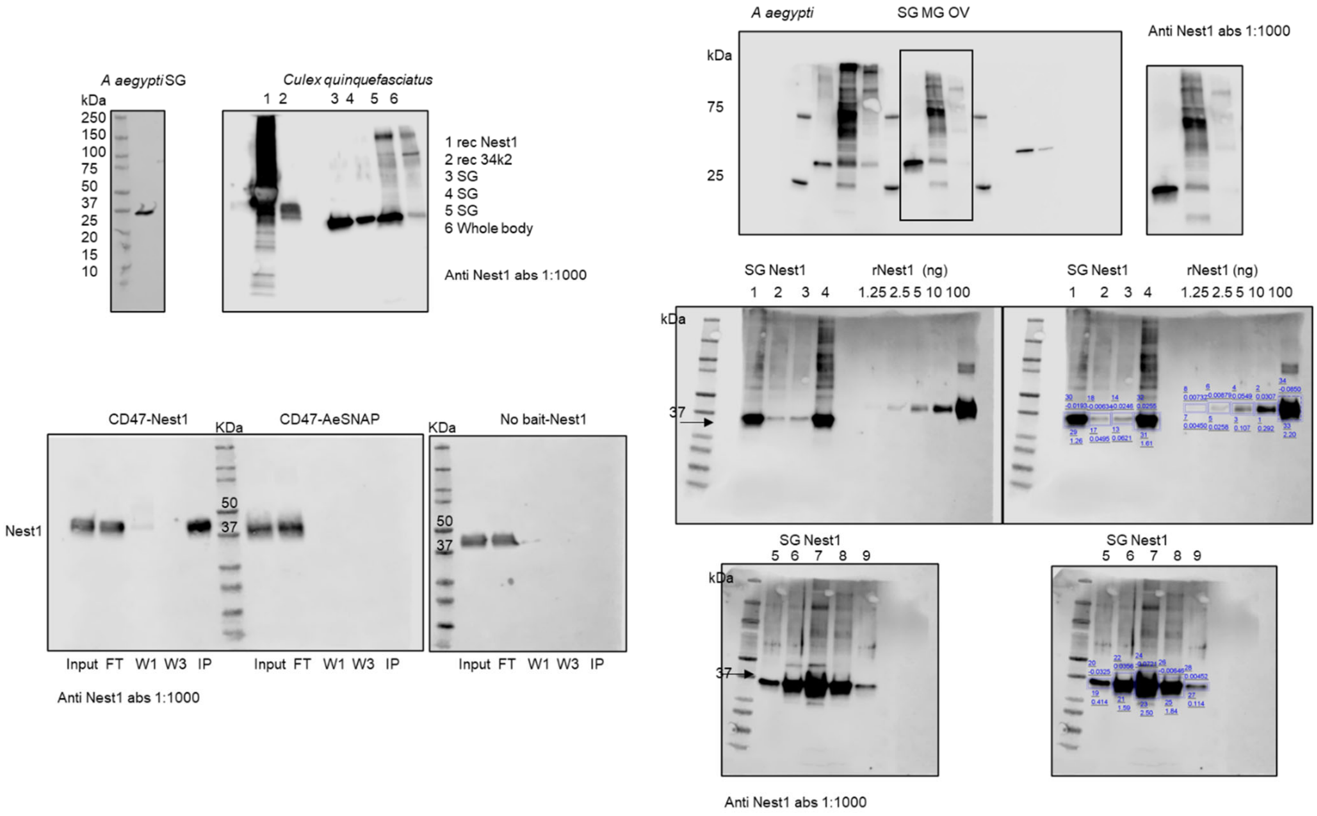

Supplementary Figure 8. Original unmodified protein immunoblots.

Gene	Forward sequence	Reverse sequence
IFNγ	GAGTGTGGAGACCATCAAGGAAG	TGCTTTGCGTTGGACATTCAAGTC
IL-2	AGAACTCAAACCTCTGGAGGAAG	GCTGTCTCATCAGCATATTCACAC
TNF	CTCTTCTGCCTGCTGCACTTTG	ATGGGCTACAGGCTTGTCACTC
IL-12p40	GACATTCTGCGTTCAGGTCCAG	CATTTTTGCGGCAGATGACCGTG
IL-8	GAGAGTGATTGAGAGTGGACCA	CACAACCCTCTGCACCCAGTTT
IL-6	AGACAGCCACTCACCTCTTCAG	TTCTGCCAGTGCCTCTTTGCTG
IL-13	ACGGTCATTGCTCTCACTTGCC	CTGTCAGGTTGATGCTCCATACC
IL-4	CCGTAACAGACATCTTTGCTGCC	GAGTGTCCTTCTCATGGTGGCT
IL-5	GGAATAGGCACACTGGAGAGTC	CTCTCCGTCTTTCTTCTCCACAC
IL-1RA	ATGGAGGGAAGATGTGCCTGTC	GTCCTGCTTTCTGTTCTCGCTC
IL-10	TCTCCGAGATGCCTTCAGCAGA	TCAGACAAGGCTTGGCAACCCA
MCP-1	AGAATCACCAGCAGCAAGTGTCC	TCCTGAACCCACTTCTGCTTGG
GAPDH	GGATTTGGTCGTATTGGG	GGAAGATGGTGATGGGATT
Nest1	ACTGGAGACGAACTACCGGA	TTCCTGCCGTTCTCCTTGAC
Rp49	GCTATGACAAGCTTGCCCCCA	TCATCAGCACCTCCAGCT
ZIKV MEX2–81	TTGGTCATGATACTGCTGATTGC	CCTTCCACAAAGTCCCTATTGC
ZIKV MEX-I44 1086	CCGCTGCCCAACACAAG	
ZIKV MEX-I44 1062c		CCATCAACGTTCTTTTGCAGACAT
ZIKV 1107-FAM(IFQ)	AGCCTACCTTGACAAGCAGTCAGACACTCAA	

Supplementary table 1. qRT-PCR primer sequences used to detect human, *Aedes* and ZIKV transcripts.
